# Bonobo anatomy reveals stasis and mosaicism in chimpanzee evolution, and supports bonobos as the most appropriate extant mode﻿l for the common ancestor of chimpanzees and humans

**DOI:** 10.1038/s41598-017-00548-3

**Published:** 2017-04-04

**Authors:** Rui Diogo, Julia L. Molnar, Bernard Wood

**Affiliations:** 1 Department of Anatomy, Howard University College of Medicine, Washington, DC USA; 2 CASHP, Department of Anthropology, George Washington University, Washington, DC USA

## Abstract

Common chimps and bonobos are our closest living relatives but almost nothing is known about bonobo internal anatomy. We present the first phylogenetic analysis to include musculoskeletal data obtained from a recent ﻿dissection of bonobos. Notably, chimpanzees, and in particular bonobos, provide a remarkable case of evolutionary stasis for since the chimpanzee-human split c.8 Ma among >120 head-neck (HN) and forelimb (FL) muscles there were only four minor changes in﻿ the chimpanzee clade, and all were reversions to the ancestral condition. Moreover, since the common chimpanzee-bonobo split c.2 Ma there have been no changes in bonobos, so with respect to HN-FL musculature bonobos are the better model for the last common ancestor (LCA) of chimpanzees/bonobos and humans. Moreover, in the hindlimb there are only two muscle absence/presence differences between common chimpanzees and bonobos. Puzzlingly, there is an evolutionary mosaicism between each of these species and humans. We discuss these data in the context of available genomic information and debates on whether the common chimpanzee-bonobo divergence is linked to heterochrony.

## Introduction

In the past decade researchers published the draft sequences of the nuclear genomes of common chimpanzees^1^, orangutans^2^, gorillas^3^ and bonobos^4^. Since these initial publications better quality data and larger data sets have become available, and recently the publication of an additional 40 complete common chimp/bonobo genomes with a 25-fold sequence coverage has clarified both the timing of the split, and the patterns of subsequent gene exchange, between these two species^5^. Such genomic evidence provides a comparative framework for understanding the evolutionary time scale of the phenotypic differences among extant apes and between the latter and humans. Therefore, in the last years we have carried out systematic dissections of cadavers of most extant primate taxa to gather evidence about how soft tissue - in particular striated muscle - gross morphology differs among living primates, with a focus on the great apes. The initial dissections collected evidence about the muscles of the head and neck (HN) and forelimb (FL)^6, 7^ and more recently we have added information about the hindlimb (HL) and trunk^8–12^. The data collected range from the presence or absence of individual muscles to more detailed observations about their morphology (e.g. numbers of muscle bellies), attachments (e.g. which digits they attach to) and innervation. These data were used to generate characters that were used to reconstruct relationships among the taxa sampled^6, 7^ and to undertake the first comparison of morphological (muscle) *vs*. genetic evolutionary rates of change among different branches of the primate clade^13^.

The lineages leading to modern humans (hominins) and to common chimps/bonobos (panins) separated *c*.8 million years ago (Mya), while common chimpanzees and bonobos separated *c*.2 Mya. Given the reasonable assumption that the gross morphology of muscles is related to the profound differences in posture, locomotion and dexterity between modern humans and common chimpanzees and bonobos, it is crucial to explore the implications of the pattern of muscle differences that have accumulated in the hominin and panin lineages in the past 8 Ma. However, until very recently comprehensive data about the soft tissues of panins were only available for common chimpanzees - a previous study of bonobo musculature was incomplete and restricted to a single individual^14^. Few zoos keep bonobos and cadavers are difficult to come by, but thanks to the foresight of researchers at the Antwerp Zoo, which has one of the largest collections of bonobos in captivity, seven bonobo cadavers - six fresh (frozen) and one preserved in formalin – had been preserved. Thanks also to a collaboration between the Antwerp Zoo and the Applied Veterinary Morphology group of the Department of Veterinary Sciences at the University of Antwerp, arrangements were made for a team of researchers to dissect all seven cadavers (including fetal, infant, adolescent, and adult individuals of both sexes) in circumstances that allowed their anatomy to be compared as the dissections progressed. In the present report we provide a first-hand summary of the most important differences between bonobos, common chimpanzees and modern humans and compare these species with other apes and primates in order to assess the broader evolutionary implications of these differences. More details about the detailed results of the seven dissections are given in the SI and in the forthcoming photographic atlas of bonobos^15^.

## Materials and Methods

The dissection of the seven bonobo (*Pan paniscus*) specimens from the Antwerp Zoo took place at the Antwerp University. All were fresh (frozen), except Lomela, which was formalin embalmed. The specimens were: Kidogo (ZIMS 164031), adult male, 35.0 Kg; Lomela (ZIMS 164046), adult female, 37.7 Kg; Jasiri (ZIMS 164047), 8 years-old adolescent female, 25.7 Kg; Etje (ZIMS 164040), 2 month-old infant male, 1.9 Kg; Foyo (ZIMS 164041), 8 month-old infant male, 2.7 Kg; Gabber (ZIMS 164042), 2 month-old infant female, 1.6 Kg; Ano (ZIMS 164052), fetus female, 0.7 Kg. The dissections were made by the members of the Bonobo Morphology Initiative (BMI) ﻿2016﻿, a team of comparative anatomists and biological anthropologists. Dissections were carried muscle by muscle, and layer by layer, taking photographs and notes about the overall configuration and attachments and innervation and then removing each muscle from superficial to deep. Details of each member of the BMI 2016 team, and their contributions, are given in our atlas of bonobos^15^.

In addition, for the comparisons with common chimpanzees, modern humans and other primates, detailed data obtained from previous dissections of many other non-bonobo primates undertaken by the authors and colleagues were also used^6–13^. This broad comparative context is crucial for establishing the homologies among the muscular structures of these taxa. It is also important to use an informed, coherent muscle nomenclature for all these taxa, which is based on that employed in modern human but also takes into account the names used by researchers who have focused on non-human primates. Regarding the phylogenetic methods used in the cladistic analysis, they were the same as those described in detail by Diogo & Wood^6, 7^. Two points should be stressed about the sample size used in the cladistic study, and the issue of anatomical variability. First, it is difficult to find primate, and particularly ape, specimens in circumstances where careful dissection can take place, as noted above. During our long-term project we made a considerable effort to establish connections with museums and zoos in the US and beyond, which enabled us to dissect a large sample of non-human primates, including apes^6, 7^. The second point is that the sample size used in the cladistic study refers to the specimens dissected by us, plus the total number of specimens reported in the numerous publications by other authors that we reviewed for this long-term project. That is, when we code each character and code a muscle present because it is found in ≥ 50% of the dissected specimens of a certain terminal taxon, we take into account all the information available. For instance, for char. 118 (the presence ⁄ absence of the palmaris longus) we take into account information obtained from dissections (by us and by others) of more than 20 hylobatid specimens, 19 orangutans, 25 gorillas, 38 common chimps and 11 bonobos, i.e. the seven bonobo specimens dissected by us plus bonobo specimens previously dissected for works of other authors (e.g., refs 14, 16–20) that were included in our literature review for the bonobo atlas^15^. So, in this specific example, for a single phylogenetic character, the total sample size, just for apes, is 123 specimens; as for most of these specimens we have information about the two sides of the body, we have information, just for apes, for >200 cases, on whether the palmaris longus was present in a limb or not. Such a total sample size is high when compared to other cladistic studies based on morphology, and particularly those based on soft tissue characters. For more details about the methodology of the phylogenetic analysis, as well as about the methodology concerning the dissections and anatomical comparisons, see Diogo & Wood^6, 7^ and Diogo *et al*.^8–12^ (the methodology used for the bonobo dissections and comparisons, given in the bonobo atlas^15^, is the same to that provided in the atlases already published^8–12^).

## Results and Discussion

**Figure 1:**
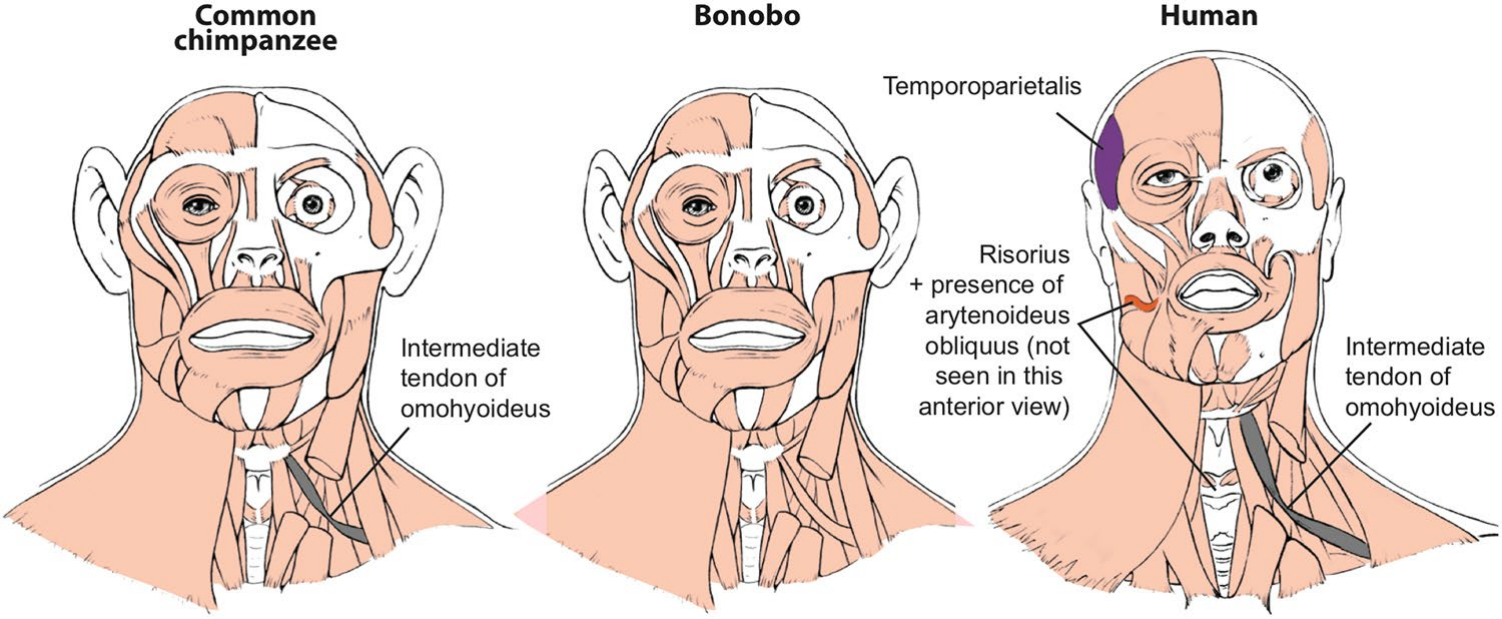
Differences between head muscles of common chimpanzees, bonobos and modern humans. There are no major consistent differences concerning the presence/absence of muscles in adult common chimpanzees (left) and bonobos (center), the only minor difference (shown in grey in the common chimpanzee scheme) being that the omohyoideus has no intermediate tendon in bonobos, contrary to common chimpanzees (and modern humans). In contrast, there are many differences between bonobos and modern humans (right) concerning the presence/absence of muscles in the normal phenotype (shown in colors and/or with labels in the human scheme). See text for more details.

**Figure 2:**
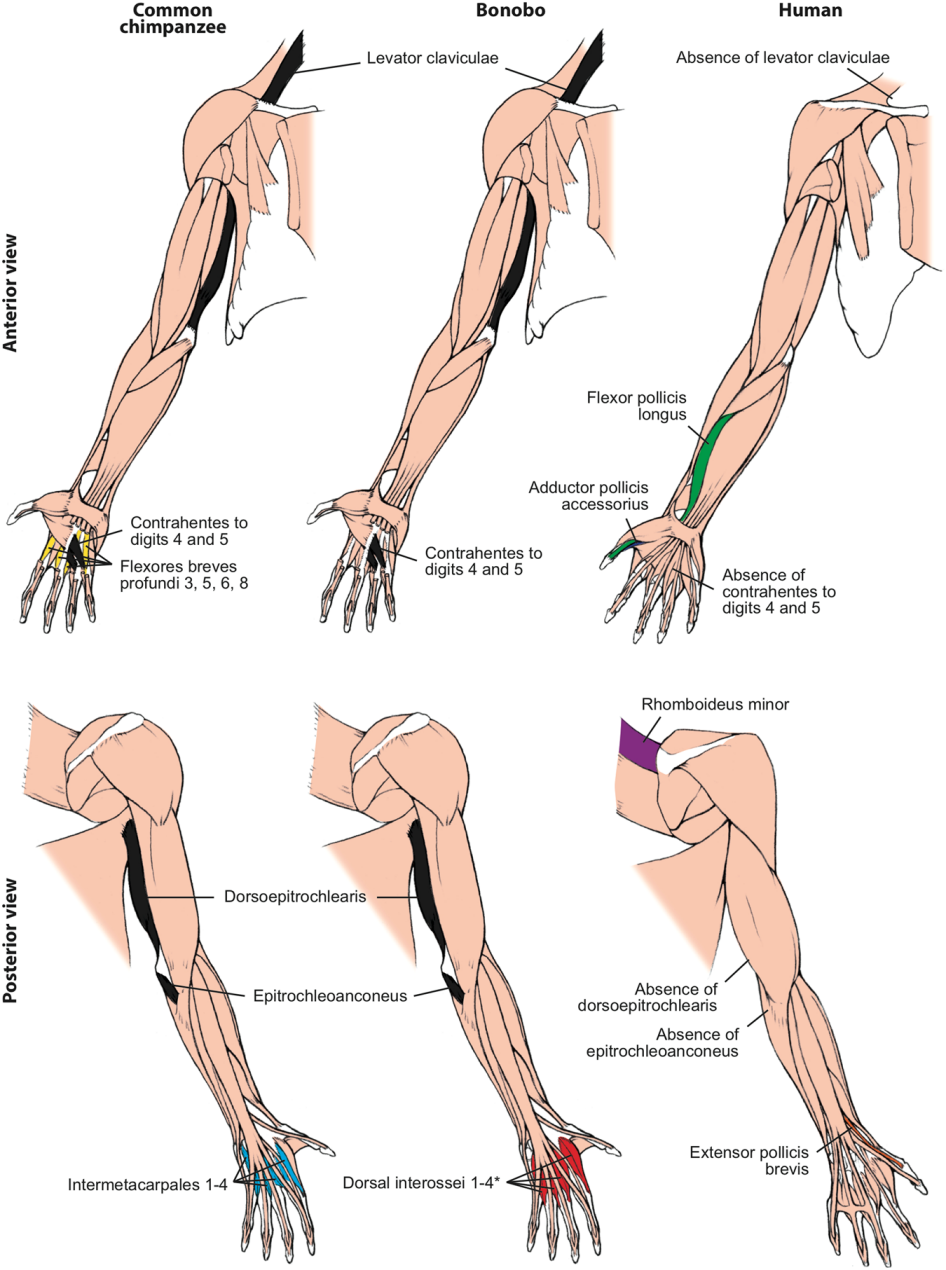
Differences between forelimb muscles of common chimpanzees, bonobos and modern humans. The only consistent difference between bonobos (center) and common chimpanzees (left) concerning the presence/absence of muscles (shown in colors in the common chimpanzee and bonobos schemes) is that in the former the intermetacarpales 1–4 are usually fused with the flexores breves profundi 3, 5, 6 and 8 to form the dorsal interossei muscles 1–4 (* in bonobo) figure, as is the case in modern humans. In contrast, there are many differences between bonobos and modern humans (right) concerning the presence/absence of muscles (shown in colors and/or with labels in the human scheme; muscles present in chimpanzees and not in humans are shown in black, in chimpanzees). See text for more details.

**Figure 3:**
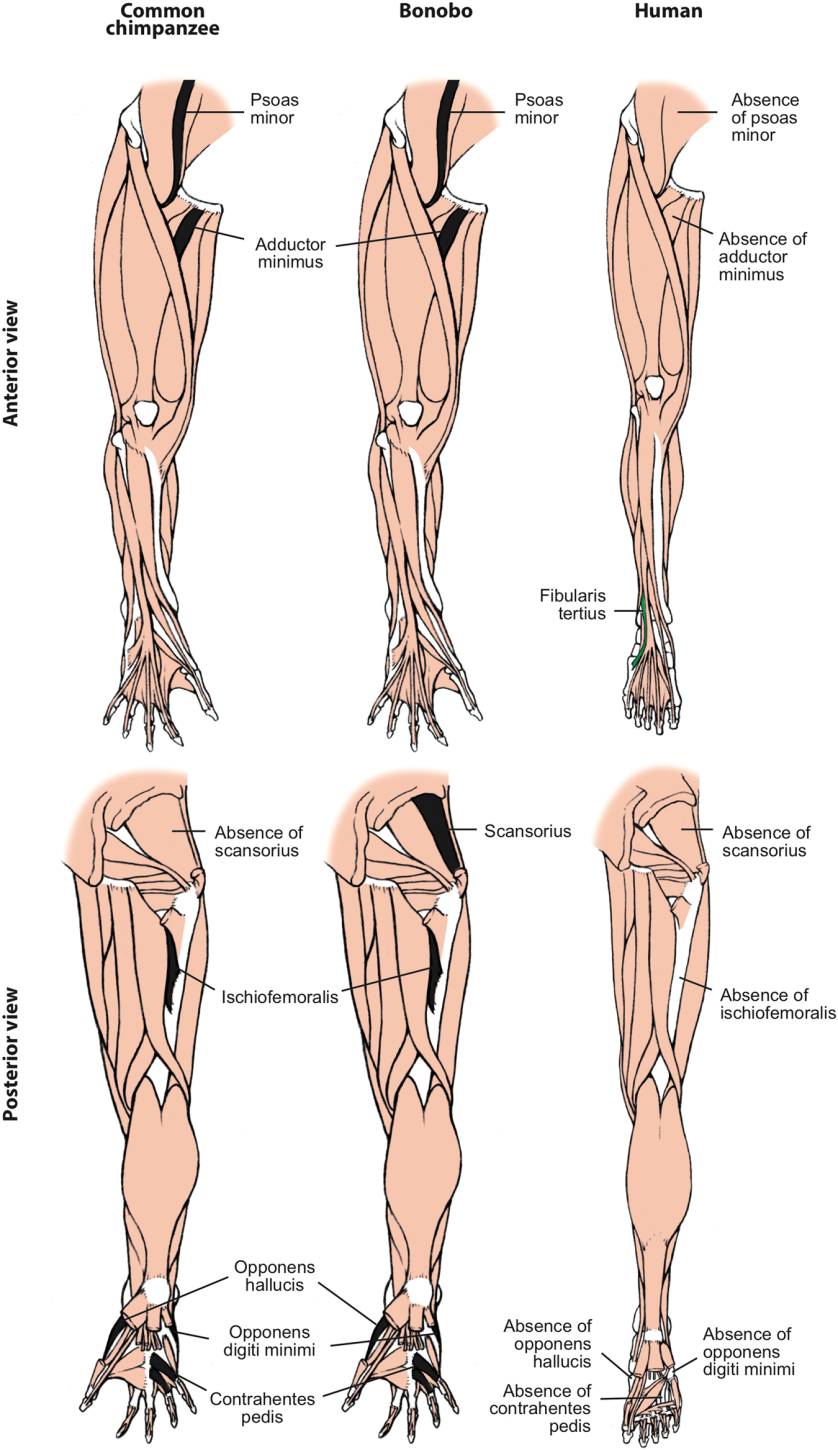
Differences between hindlimb muscles of common chimpanzees, bonobos and modern humans. The only consistent difference between bonobos (center) and common chimpanzees (left) concerning the presence/absence of muscles (shown in colors in the common chimpanzee scheme) is that the latter usually lack the scansorius, as is the case in humans. In contrast, there are many differences between bonobos and modern humans (right) concerning the presence/absence of muscles (shown in colors and/or with labels in the human scheme; muscles present in chimpanzees and not in humans are shown in black, in chimps). See text for more details.

Among the 166 HN and FL muscle characters recognized in our earlier phylogenetic studies^6, 7^, there were 16 character state differences between common chimpanzees and modern humans (Figs 1 and 2; see SI). The new HL data added 12 more differences: the usual absence in modern humans of a psoas minor, ischiofemoralis, adductor minimus, opponens hallucis, contrahentes pedis, opponens digiti minimi, and of tendons of flexor hallucis longus to digits 3–4, and the presence of a fibularis tertius, of digit 2 as the interossei axis of the foot, and of fibularis longus-medial cuneiform, soleus-tibia and flexor digitorum brevis-digit 5 attachments (see Fig. 3 and refs 11, 15). Therefore, since the chimpanzee-human split *c*.8 Mya, the divergence rate for striated muscle gross morphology has been *c*.3.5 characters per million years (Ma). In comparison, across the body there are seven differences in muscle morphology between common chimpanzees and bonobos (Figs 1 and 3; SI). This divergence rate of *c*.3.5 muscle characters per Ma since common chimpanzees and bonobos split *c*.2 Ma is similar to the divergence rate for common chimpanzees and modern humans. Interestingly, only one of the common chimpanzee/bonobo differences involves HN muscles: bonobos have a single belly of the omohyoideus, compared to the two bellies that are usually present in both common chimpanzees and modern humans (Fig. 1). Three of the differences are in the FL: (1) the intermetacarpales and flexores breves profundi muscles in the hand of bonobos fuse to form dorsal interossei, a shared feature with modern humans; (2) bonobos have a stout tendon of the flexor digitorum profundus attaching to digit 1, and (3) an attachment between the pectoralis minor and the coracoid process of the scapula (Fig. 2). In the HL, bonobos retain a scansorius and have popliteus-fibula and extensor hallucis longus-proximal big toe phalanx attachments: all these features are missing in common chimpanzees and modern humans (Fig. 3).

Importantly, of the seven common chimpanzee/bonobo differences only two, the presence of dorsal interossei and scansorius in bonobos, concern major differences (i.e., presence/absence of muscles). This contrasts with the 20 major differences between common chimps and modern humans (13 for HN-FL + 7 for HL). The seven common chimpanzee modern human HL muscle absence/presence differences were listed within the 12 HL differences listed in paragraph above. The 13 HN-FL differences are: the presence in modern humans of the HN muscles temporoparietalis, risorius and arytenoideus obliquus (these two latter muscles are only present as variants in chimpanzees) and of the FL muscles rhomboideus minor, flexor pollicis longus, adductor pollicis accessorius, extensor pollicis brevis; presence in common chimpanzees of the FL muscles levator claviculae, dorsoepitrochlearlis, epitrochleoanconeus, contrahentes to digits 4 and 5, and intermetacarpales (Figs 1, 2 and 3 and refs 11, 15). The number of major bonobo-human muscle absence/presence differences is exactly the same, i.e. 13, because bonobos and modern humans do not have distinct intermetacarpales (Fig. 2), but bonobos are different from modern humans in having a scansorius (Fig. 3). Thus, although the overall common chimpanzee/bonobo *vs*. modern human, and common chimpanzee *vs*. bonobo divergence rates are similar overall, in terms of major changes the common chimpanzee *vs*. bonobo divergence rate (two character state differences in *c*.2.0 Ma) is strikingly (>2.5 times) lower than the common chimpanzee *vs*. modern human divergence rate of 20 major changes in *c*.8 Ma. The rate for the bonobo *vs*. modern human divergence is also higher (>1.6 times) than the bonobo vs. common chimp divergence concerning major changes, because there are also 13 major differences between the musculature of bonobos and modern humans. This is because in the FL bonobos and modern humans have dorsal interossei in contrast to common chimpanzees (Fig. 2), but in the HL bonobos differ from modern humans and common chimpanzees because they retain a scansorius (Fig. 3). Remarkably, all seven differences between common chimpanzees and bonobos are features that are shared by one of the two panin species and modern humans. Moreover, the analysis of these differences reveals a *mosaic evolution* across the three lineages. Different anatomical regions are evolving in markedly different ways in different taxa: the four features shared between common chimpanzees and modern humans concern the HN and HL muscles, whereas the three bonobo-modern human similarities concern the FL muscles. It will be interesting to investigate whether the four former features are under the control of the approximately three per cent of the modern human genome that is more closely related to the common chimpanzees, and the three later features are linked with the three per cent of our genome that is shared with the genome of bonobos^4^.

**Figure 4:**
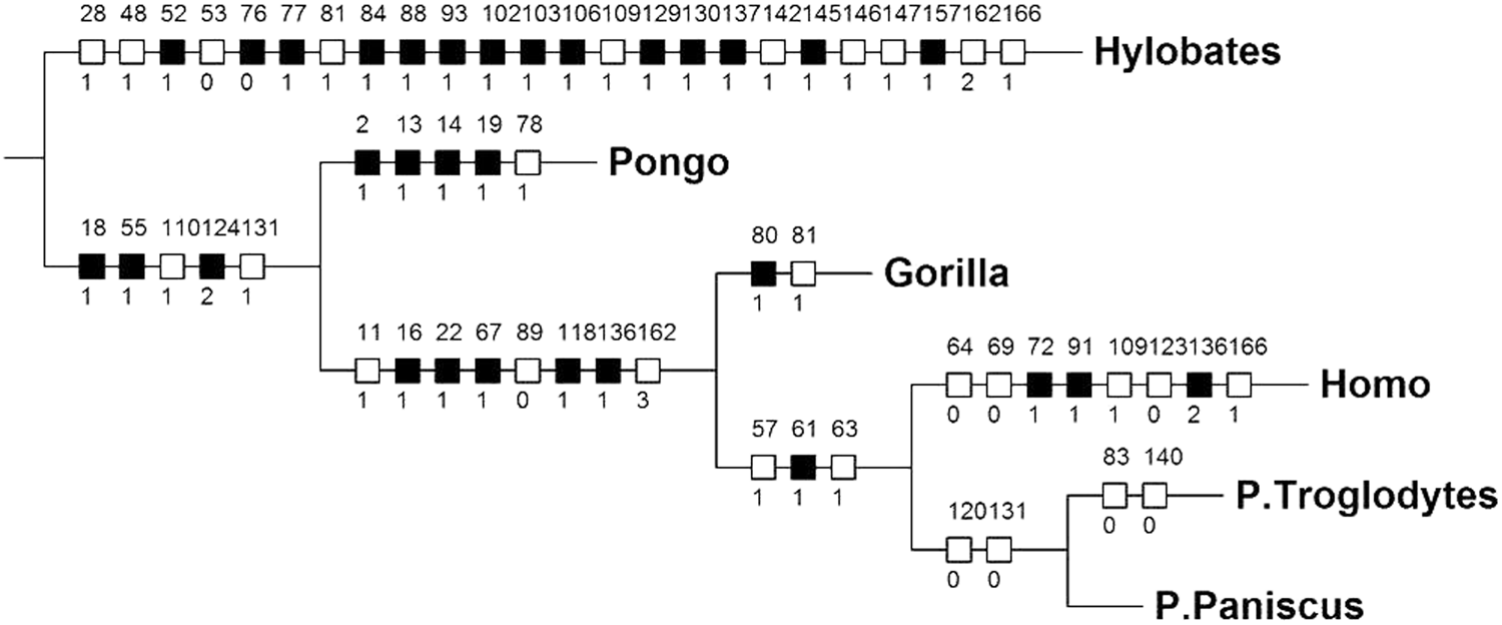
Cladogram showing evolutionary changes in head-forelimb musculature in hominoids. Single most parsimonious tree (L = 303, CI = 57, RI = 75) obtained in our phylogenetic analysis; for a key of the characters and character state changes show in the cladogram, see text and SI. Note how node leading to LCA of two *Pan* species has only two changes; *P*. *troglodytes* then accumulated two changes, with no changes in the bonobo lineage (for more details, see text). Note that the phylogenetic software converts *P. paniscus* and *P. troglodytes* into *P. Paniscus and P. Troglodytes.*

The relatively small number of changes in striated muscle morphology in the panin clade compared to the human clade is particularly evident when the data for *P*. *paniscus* are included in a phylogenetic analysis of the 166 HN and FL muscle characters used in our previous studies^6, 7^ (Fig. 4 and SI). Importantly, the inclusion of these data affected the distribution of synapomorphies at two thirds (6/10) of the nodes in the tree (Fig. 4), including the one leading to modern humans, relative to the tree obtained in our previous analyses (see refs 6, 7). Firstly, there was a removal of the 0 –> 1 change of char. 112 (CS1 vestigial tendon of long flexor to distal phalanx of digit 1) from the node leading to great apes + humans and of the 1 –> 0 change of this character from the human lineage, as the presence of a non-vestigial tendon in bonobos now makes it equally parsimonious to have CS1 acquired in great apes + humans and then reverted in humans and bonobos or an independent acquisition of CS1 in common chimpanzees, gorillas and orangutans (3 steps). Secondly, the 0 –> 1 change in char. 66 (CS1 intermediate tendon of omohyoideus present) was removed in the node leading to panins + humans, as the absence of tendon in bonobos now makes it equally parsimonious to have acquisition of the tendon in this node and subsequent loss in bonobos or an independent acquisition in *P*. *troglodytes* and modern humans (2 steps). Thirdly, two of the features that were previously seen as *Pan* synapomorphies (concerning char. 140: CS1 intermetacarpales not present as separate muscles; and char. 83: CS1 pectoralis minor going to coracoid process) are now assigned to *P*. *troglodytes*, i.e. reversion to CS0 in both characters. Thus, the *Pan* clade thus now has only two synapomorphies (reversion to CS0 in char. 120: CS1 not having a distinct epitrochleoanconeus; and in char. 131: CS1 contrahentes digitorum missing). These changes emphasize the importance of taking information about bonobos into account in studies on human evolution.

As can be seen in Fig. 4, the rate of HN and FL muscle evolutionary changes in the human clade (4 in 8 Ma, i.e. 0.5 per Ma) is twice that in the chimpanzee clade (8 in 8 Ma, i.e. 1 per Ma). Moreover, all the four changes in the chimpanzee clade are reversions to the ancestral state, i.e. there is not even a single acquisition of a derived muscle feature within that clade, whereas in the human clade there are several examples of muscles that are not part of the normal phenotype in any other primate (i.e., they are autapomorphies). A similar picture also applies to the striated musculature of the hindlimb, in that all of the 12 differences in HL muscles between common chimpanzees/bonobos *vs*. modern humans are due to derived changes in the lineage leading to modern humans (Fig. 3)^15^. Furthermore, since the split between common chimpanzees and bonobos *c*.2 Mya the only two changes that occurred in the chimpanzee clade are within the lineage leading to common chimpanzees. That is, within all the 124 HN and FL muscles of bonobos (Table S2) there is not a single minor change - even including a simple site of origin or attachment, or a fusion, of a muscle - in the 2 Ma of evolution of the bonobo lineage. This is a striking example of *evolutionary stasis*.

To put these results in perspective, it is useful to compare them with those of our previous paper focusing specifically on the comparison of primate morphological (muscle) and molecular evolutionary rates^13^. This is because the phylogenetic analysis, terminal taxa and muscle characters that were used as a basis for that paper are exactly the same as those used for the present paper, with the difference that we now include bonobos as an additional terminal taxon (see SI and Fig. 4). It should be noted that for the earlier paper the muscle rates were calculated taking into account both the split between clades and the time of appearance of each terminal taxon (genus) calculated by taking into account genetic data available for various species within a genus. Figure 4 of this genus (as they are shared by different gorilla species) were assumed to have been acquired during 7.1 Ma, i.e. a rate of 0.28 (2/7.1) changes per Ma. Using the same methodology, in that paper the muscle rate leading to the genus *Pongo* was 0.51 changes per Ma. As explained in detail in that paper, these muscle evolutionary rates are very slow when compared with the rates leading to other hominoid terminal taxa such as *Hylobates* (rate of 2.72) and *Homo* (rate of 1.77; for more details, see that paper). So, if we were to use the same methodology in order to compare those rates in a systematic way with the results obtained for chimpanzees in the cladistic analysis of the present paper and thus set the chimpanzee/human split at 7.5 Mya and the origin of the genus *Pan* at *c.* 3 Mya as we did in that paper, instead of using respectively the 8 Mya and 2 Mya dates suggested in more recent genetic studies (see above) the rate leading to *Pan* in the cladogram of Fig. 4 would be only 4.4 muscle changes per Ma (2 muscle changes in 4.5 MMa). This results in a rate even lower than the very slow rate of 0.51 leading to the genus *Pongo*, for instance. Moreover, within all the hominoid genera, *Pan* is the only one that lacks a unique feature/muscle character state - or even a derived character - as a synapomorphy. The only two muscles changes acquired are reversions to the plesiomorphic state and, even more remarkably, there we no muscle changes, major or minor, leading to *P. paniscu﻿s*, as noted above.

These new bonobo data are also relevant to two major ongoing debates in biological sciences. The first is whether bonobos or common chimpanzees are a better model for the last common ancestor (LCA) of chimpanzees, and of chimpanzees and modern humans. Zihlman and colleagues have suggested that, within the two extant chimpanzee species, bonobos make a more suitable model for the two LCAs^21–24^, but others have proposed that bonobos are highly derived chimpanzees, being for instance adapted to unique ecological conditions that selected for a specialized locomotor habits^25^. Our data do not support the latter suggestion because bonobos do not display a single muscle or muscle feature that is unique within primate, or even hominoid, evolution. In fact, it is now becoming increasingly accepted that the bonobo-common chimpanzee divergence was likely mainly due to the barrier to gene flow created by the formation of the Congo River *c*.1.5–2.5 Mya. Since then relatively little hybridization has occurred between bonobos and common chimpanzees on opposite sides of this river^4^. This scenario can therefore help to explain why the anatomical differences between the two *Pan* species are so minor when compared to the striking anatomical differences between them and humans. The second debate is about hypothesized differences in the ontogenetic trajectories of common chimpanzees and bonobos. Some have argued that bonobos are more paedomorphic than chimpanzees^26^, but more recently Bolter and Zihlman^27^ suggested that skeletal development in common chimpanzees is slower than that of bonobos, an idea also partially supported in a recent study linking phenotypic development and genotypic variation in common chimpanzees and bonobos^28^. One of the two major (presence/absence) muscle differences between these two species is consistent with the hypothesis that the musculoskeletal development of common chimps is slower than that of bonobos. This is because early in human ontogeny the intermetacarpales are distinct muscles (as they remain in adult common chimpanzees), and only in the later stages of human development do they become fused with the flexores breves profundi to form the dorsal interossei (as they are in adult bonobos)^29, 30^. These discussions emphasize how the study of the soft tissues of bonobos and other apes is crucial for a comprehensive and integrative understanding of the evolution and biology of chimpanzees, other apes, and primates, and ultimately of our own human lineage.

## Electronic supplementary material

Supplementary Information
