# Supplementary material for: Bonobo anatomy reveals stasis and mosaicism in chimpanzee evolution, and supports bonobos as the most appropriate extant mode﻿l for the common ancestor of chimpanzees and humans
Source: Sci Rep. 2017 Apr 4;7:608. doi: 10.1038/s41598-017-00548-3 (PMC5428693; doi:10.1038/s41598-017-00548-3)
Supplement: Supplementary file 1 — Supplementary Information [file 41598_2017_548_MOESM1_ESM.doc]

**First detailed bonobo anatomy study reveals striking static and mosaic chimpanzee evolution and bonobos as best model for human-chimpanzee ancestor**

**Authors:** Rui Diogo, Julia L. Molnar, Bernard Wood

**Supplementary Materials**

List of 166 phylogenetic characters including *P. paniscus*

Table S1: matrix of 166 phylogenetic characters including *P. paniscus*

Table S2: table showing total number of HN-FL muscles in primates, including *P. paniscus*

**List of 166 HN and FL characters included in the cladistic analysis**

This brief summary of the list of the 166 phylogenetic characters used by Diogo & Wood ^2-3^ is divided into nine subgroups of muscles, and includes, for the first time, *Pan paniscus* as a terminal taxon (for moe details, see text). The Length (L), Consistency Index (CI) and Retention Index (RI) obtained for each character in the most parsimonious tree obtained from the heuristic analysis of the 166 characters are given after the name of each respective character.

**Mandibular muscles**

1. ***Intermandibularis anterior*** *is not a distinct muscle (L 1, CI 100, RI 100)*. Contrary to taxa of CS-0 **[0]**, in *Cynocephalus* and the primates included in this study **[1]** there is usually no fleshy, separate intermandibularis anterior.
2. ***Digastricus anterior*** *is not a distinct muscle (L 1, AUTAPOMORPHY)*. Contrary to taxa of CS-0 **[0]**, in *Pongo* **[1]** the digastricus anterior is usually not present as a distinct muscle.
3. ***Digastricus anterior*** *is not in contact with its counterpart for most of its length (L 6, CI 16, RI 37)*. Contrary to taxa of CS-0 **[0]**, in *Cynocephalus*, *Lemur*, *Propithecus*, *Loris*, *Nycticebus, Aotus, Callithrix*, *Pithecia*, *Colobus*, *Cercopithecus*, *Hylobates* and *Homo* **[1]** the digastricus anterior does not contact its counterpart for most of its length. **[-]** Inapplicable in *Pongo*, because the digastricus anterior is usually not present as a distinct muscle.
4. ***Digastricus anterior*** *is not connected to the* ***digastricus posterior*** *by a well-defined intermediate tendon (L 1, AUTAPOMORPHY)*. Contrary to taxa of CS-0 **[0]**, in *Cynocephalus* **[1]** the digastricus anterior and digastricus posterior are not connected by a well-defined tendon, but instead by a short muscular intersection. **[-]** Inapplicable in *Pongo*, because the digastricus anterior is usually not present as a distinct muscle.
5. ***Digastricus anterior*** *attaches onto the angle of the mandible (L 1, AUTAPOMORPHY)*. Contrary to taxa of CS-0 (in which the digastricus anterior usually inserts onto and/or near the mandibular symphysis) **[0]**, in *Cynocephalus* **[1]** the digastricus anterior usually inserts onto the angle of the mandible. **[-]** Inapplicable in *Pongo*, because the digastricus anterior is usually not present as a distinct muscle.
6. ***Tensor tympani*** *is not a distinct muscle (L 1, AUTAPOMORPHY)*. Contrary to taxa of CS-0 **[0]**, in *Tupaia* **[1]** the tensor tympani is usually not present as a distinct muscle. *Colobus*, *Cercopithecus* and *Pongo* are coded as “?”.
7. *Chorda tympani passes above the* ***tensor tympani*** *(L 1, CI 100, RI 100)*. In *Rattus*, *Cynocephalus*, *Lemur*, *Propithecus*, *Loris* and *Tarsius* **[0]**, the chorda tympani passes mainly below the tensor tympani (hypotensoric). In *Aotus*, *Callithrix*, *Saimiri*, *Pithecia*, *Macaca*, *Papio*, *Hylobates*, *Gorilla*, *Pan paniscus, Pan troglodytes* and *Homo* **[1]**, it passes mainly above the muscle (epitensoric). *Nycticebus*, *Colobus*, *Cercopithecus* and *Pongo* are coded as “?”. **[-]** Inapplicable in *Tupaia*, because the tensor tympani is usually not present as a distinct muscle.
8. ***Temporalis*** *has a pars suprazygomatica (L 3, CI 33, RI 66)*. Contrary to taxa of CS-0 **[0]**, in *Tupaia, Lemur, Propithecus, Loris, Nycticebus, Tarsius, Aotus, Callithrix*, *Saimiri, Pithecia, Colobus, Cercopithecus,* and *Papio* **[1]** there is a distinct pars suprazygomatica of the temporalis. *Macaca* and *Gorilla* are coded here as "?".
9. ***Pterygoideus lateralis*** *has well differentiated inferior and superior heads* *(L 2, CI 50, RI 85)*. Contrary taxa of CS-0 **[0]**, in *Aotus*, *Pithecia*, *Saimiri*, *Macaca*, *Papio, Colobus, Cercopithecus, Hylobates*, *Pongo*, *Gorilla, Pan paniscus, Pan troglodytes* and *Homo* **[1]** the pterygoideus lateralis is usually well differentiated into distinct superior and inferior heads. *Loris* and *Nycticebus* are coded as “?”.

**Hyoid muscles**

1. ***Stylohyoideus*** *is not a distinct muscle (L 2, CI 50, RI 50)*. Contrary to taxa of CS-0 **[0]**, in *Cynocephalus, Saimiri* and *Callithrix* **[1]** the stylohyoideus is usually not present as a distinct muscle.
2. ***Stylohyoideus*** *is partially pierced by the* ***digastricus posterior*** *and/or by the intermediate digastric tendon (L 3, CI 33, RI 66)*. Contrary to taxa of CS-0 **[0]**, in *Tupaia*, *Cercopithecus*, *Papio, Colobus*, *Gorilla*, *Pan paniscus, Pan troglodytes* and *Homo* **[1]** the stylohyoideus is usually partially pierced by the digastricus posterior and/or by the intermediate digastric tendon. *Macaca* and *Pithecia* are coded as "?". **[-]** Inapplicable in *Cynocephalus, Saimiri* and *Callithrix* because the stylohyoideus is usually not present as a distinct muscle.
3. ***Stylohyoideus*** *is inserted near the midline* *(L 1, CI 100, RI 100)*. Contrary to taxa of CS-0 **[0]**, in *Propithecus* and *Lemur***[1]** the distal insertion of the stylohyoideus onto the hyoid bone is peculiarly situated near the midline (i.e., the muscle almost reaches, or sometimes even contacts, its counterpart medially). **[-]** Inapplicable in *Cynocephalus, Saimiri* and *Callithrix* because the stylohyoideus is usually not present as a distinct muscle.
4. ***Stylolaryngeus*** *is a distinct muscle (L 1, AUTAPOMORPHY)*. Contrary to taxa of CS-0 **[0]**, orangutans **[1]** usually have a distinct stylolaryngeus muscle that runs from the styloid process to the laryngeal sac.
5. ***Digastricus posterior*** *is directly attached onto the mandible (L 1, AUTAPOMORPHY)*. Contrary to taxa of CS-0 (in which the anterior portion of the digastricus posterior is usually connected to the posterior portion of the digastricus anterior) **[0]**, in *Pongo* **[1]** the anterior portion of the digastricus posterior is usually directly attached onto the back of the mandible.
6. ***Jugulohyoideus*** *is not a distinct muscle (L 2, CI 50, RI 80)*. Contrary to taxa of CS-0 **[0]**, in *Rattus*, *Tarsius*, *Aotus*, *Callithrix*, *Pithecia*, *Saimiri*, *Macaca, Colobus, Papio, Cercopithecus* and hominoids **[1]**, the jugulohyoideus (often designated as 'mastoideostyloideus', mainly running from the mastoid process and/or adjacent regions to the hyoid apparatus and/or the ligaments connecting this apparatus to the cranium) is usually not present as a distinct muscle. *Loris* is coded as "?".
7. ***Platysma cervicale*** *is not a distinct muscle (L 1, CI 100, RI 100)*. Contrary to taxa of CS-0 **[0]**, in *Pan paniscus, Pan troglodytes*, *Homo* and *Gorilla* **[1]** the platysma cervicale is usually markedly reduced or completely missing.
8. ***Platysma myoides*** *is divided into a superior, superficial bundle, and an inferior, deep bundle (L 1, CI 100, RI 100).* Contrary to taxa of CS-0 **[0]**, in *Macaca*, *Papio* and *Cercopithecus* **[1]** the platysma myoides is mainly divided into a superior, superficial bundle and an inferior, deeper bundle, which are essentially separated by a well-developed cheek pouch.
9. *‘Cervico-auriculo-occipitalis’ is not a distinct bundle of the* ***occipitalis*** *(L 1, CI 100, RI 100).* Contrary to taxa of CS-0 **[0]**, in *Homo*, *Pan paniscus, Pan troglodytes*, *Pongo* and *Gorilla* **[1]** the occipitalis is usually not differentiated into a main body (or ‘occipitalis proprius’) and a ‘cervico-auriculo-occipitalis’ (which is a lateral/superficial bundle of the occipitalis that often runs anterolaterally from the occipital region to the posterior portion of the ear and that sometimes covers part of the auricularis posterior in lateral view).
10. ***Auricularis posterior*** *is not a distinct muscle (L 1, AUTAPOMORPHY)*. Contrary to taxa of CS-0 **[0]**, in orangutans **[1]** the auricularis posterior is usually not present as a distinct muscle.
11. ***Mandibulo-auricularis*** *is not a distinct muscle (L 2, CI 50, RI 83)*. Contrary to taxa of CS-0 **[0]**, in *Cynocephalus*, *Tarsius*, and the anthropoid primates included in this study **[1]** there is usually no distinct, fleshy muscle mandibulo-auricularis.
12. ***Zygomatico-auricularis*** *is a distinct muscle (L 1, AUTAPOMORPHY)*. Contrary to taxa of CS-0 **[0]**, in *Tarsius* **[1]** the zygomatico-auricularis is present as a distinct muscle.
13. ***Risorius*** *is a distinct muscle (L 1, CI 100, RI 100)*. Contrary to taxa of CS-0 **[0]**, in modern humans, *Gorilla* and *Pan paniscus, Pan troglodytes* **[1]** the risorius is often present as a distinct muscle.
14. ***Sphincter colli superficialis*** *is not a distinct muscle* *(L 1, CI 100, RI 100)*. Contrary to *Rattus* and *Tupaia* **[0]**, in *Cynocephalus* and the primates included in this study **[1]** the sphincter colli superficialis is usually not present as a distinct muscle.
15. ***Sphincter colli profundus*** *is not a distinct muscle (L 4, CI 25, RI 66)*. Contrary to taxa of CS-0 **[0]**, in *Propithecus*, *Pithecia*, *Macaca*, *Papio*, *Colobus* and hominoids **[1]** the sphincter colli profundus is usually not present as a distinct muscle.
16. ***Sternofacialis*** *is not a distinct muscle (L 1, CI 100, RI 100)*. Contrary to *Rattus* **[0]**, in *Tupaia*, *Cynocephalus*, and the primates included in this study **[1]** the sternofacialis is usually not present as a distinct muscle.
17. ***Interscutularis*** *is not a distinct muscle (L 1, CI 100, RI 100)*. Contrary to *Rattus* **[0]**, in *Tupaia*, *Cynocephalus*, and the primates included in this study **[1]** the interscutularis is usually not present as a distinct muscle.
18. *'****Zygomaticus****' is the only well developed zygomatic muscle in the cheek region (L 1, CI 100, RI 100)*. *Callithrix, Aotus, Saimiri* and *Pithecia* **[1]**, usually have a derived condition that is not found in taxa of CS-0 **[0]**: they only have a well developed 'zygomaticus' muscle in the cheek region (and not two, i.e. a zygomaticus major and a zygomaticus minor).
19. *Anterior portion of* ***zygomaticus major*** *passes partially or completely deep to the* ***levator anguli oris facialis*** *(L 2, CI 50, RI 66)*. In taxa of CS-0 the anterior portion (i.e., the portion attaching on the angle of the mouth) of the zygomaticus major or of the lower part of the 'zygomaticus' (in New World monkeys) is superficial to the levator anguli oris facialis **[0]**. In *Cercopithecus*, *Macaca*, *Papio* and *Hylobates* **[1]** it usually passes at least partially deep to this latter muscle.
20. ***Zygomaticus major*** *is almost completely covered by the* ***platysma myoides*** *and/or the* ***platysma cervicale*** *(L 1, AUTAPOMORPHY)*. **[0]** In taxa of CS-0 the zygomaticus major (or the lower portion of the 'zygomaticus' in New World monkeys) and the platysma (myoides and/or cervicale) essentially lie at the same level or the former is partially/completely superficial to the latter, or, also often, the former lies mainly superiorly to the latter. In *Tupaia* **[1]** the zygomaticus major is usually almost completely covered by the platysma myoides and/or the platysma cervicale in lateral view. *Tarsius* is coded as "?".
21. ***Zygomaticus minor*** *is directly originated from the ear (L 5, CI 20, RI 42)*. Contrary to taxa of CS-0 **[0]**, in *Tupaia, Aotus, Callithrix, Propithecus, Lemur, Nycticebus, Loris* and *Tarsius* **[1]** the zygomaticus minor (or the upper portion of the 'zygomaticus', in New World monkeys) is directly originated from the ear.
22. ***Zygomaticus major*** *is not directly originated from the ear (L 1, CI 100, RI 100)*. Contrary to taxa of CS-0 **[0]**, in *Saimiri*, *Pithecia*, *Cercopithecus*, *Macaca*, *Colobus*, *Papio* and hominoids **[1]**, the zygomaticus major (or the lower portion of the zygomaticus in New World monkeys) is not directly originated from the ear. **[-]** Inapplicable in *Aotus* and *Callithrix*, because their muscle zygomaticus does reach the ear but these two taxa were already coded for that feature in the character above (within all the anthropoids included in this cladistic analysis, they are the only two genera in which there is a direct attachment onto the ear, and coding them together again in the present character would thus mean to code them together twice, although this clearly refers to a single feature, i.e., having the zygomaticus muscle attached onto the ear).
23. ***Frontalis*** *is* *a distinct muscle (L 2, CI 50, RI 50)*. Contrary to *Rattus* and *Saimiri* **[0]**, in *Tupaia*, *Cynocephalus* and the other primate taxa included in this study **[1]** the frontalis is usually present as a distinct muscle.
24. ***Auricularis superior*** *is* *a distinct muscle (L 1, CI 100, RI 100)*. Contrary to *Rattus* **[0]**, in *Tupaia*, *Cynocephalus* and the primates included in this study **[1]** the auricularis superior is usually present as a distinct muscle.
25. ***Zygomatico-orbicularis*** *is* *a distinct muscle (L 2, CI 50, RI 0)*. Contrary to taxa of CS-0 **[0]**, in *Tupaia* and *Cynocephalus* **[1]** the zygomatico-orbicularis is usually present as a distinct muscle.
26. ***Depressor supercilii*** *is a distinct muscle (L 1, CI 100, RI 100)*. Contrary to *Rattus***,** *Tupaia* and *Cynocephalus* **[0]**, in the primate taxa included in this study **[1]** the depressor supercilii is usually present as a distinct muscle.
27. ***Corrugator supercilii*** *is a distinct muscle (L 1, CI 100, RI 100)*. Contrary to *Rattus* **[0]**, in *Tupaia*, *Cynocephalus* and all primate taxa included in this cladistic analysis (except *Saimiri*, which is coded as "?") **[1]** the corrugator supercilii is usually present as a distinct muscle.
28. ***Levator labii superioris*** *runs mainly superoinferiorly from the region below the eye to the upper lip (L 2, CI 50, RI 85)*. **[0]** In the non-catarrhine taxa included in this analysis, but also in *Hylobates*, the levator labii superioris is not as markedly vertical (superoinferiorly directed) as is the case in taxa of CS-1: it mainly runs, instead, posteroanteriorly and lateromedially from the infraorbital region to the nose. **[1]** In the non-hylobatid catarrhine taxa included in this study the levator labii superioris runs mainly superoinferiorly from the infraorbital region to the upper lip, being less connected to the nose.
29. ***Depressor septi nasi*** *is a distinct muscle (L 1, CI 100, RI 100)*. Contrary to taxa of CS-0 **[0]**, in the catarrhines included in this study **[1]** the depressor septi nasi is usually present as a distinct muscle.
30. ***Depressor anguli oris*** *is a distinct muscle (L 2, CI 50, RI 88)*. Contrary to taxa of CS-0 **[0]**, in *Aotus* and the catarrhines included in this study **[1]** the depressor anguli oris is usually present as a distinct muscle.
31. ***Mentalis*** *is not a distinct muscle (L 1, AUTAPOMORPHY)*. Contrary to taxa of CS-0 **[0]**, in *Rattus* **[1]** the mentalis is usually not present as a distinct muscle.

**Branchial muscles**

1. ***Stylopharyngeus*** *originates from the stylomandibular ligament (L 1, CI 100, RI 100)*. In taxa of CS-0 **[0]** the stylopharyngeus usually originates from the cranium and from ligamentous, cartilaginous or ossified structures of the hyoid apparatus such as the stylohyal ligament. In *Callithrix* and *Saimiri* **[1]** a substantial part of the stylopharyngeus is originated from the stylomandibular ligament instead. *Pithecia* is coded as "?".
2. ***Ceratohyoideus*** *is not a distinct muscle (L 1, CI 50, RI 80)*. Contrary to taxa of CS-0 **[0]**, in hominoids and *Colobus* **[1]** the ceratohyoideus is usually not present as a distinct muscle. *Loris, Nycticebus* and *Papio* are coded as “?”.
3. ***Spinotrapezius*** *is not a distinct muscle (L 2, CI 50, RI 50)*. Contrary to *Rattus* and *Tarsius* **[0]**, in *Cynocephalus*, *Tupaia*, and the non-tarsoid primates included in this study **[1]** the spinotrapezius is not present as a distinct muscle (i.e., there is a single, continuous muscle trapezius).
4. ***Cleido-occipitalis*** *is not a distinct muscle (L 1, CI 100, RI 100)*. Contrary to *Rattus* and *Tarsius* **[0]**, in *Cynocephalus* and the primates included in this study **[1]** the cleido-occipitalis is usually not present as a distinct muscle.
5. ***Trapezius*** *inserts onto the clavicle (L 3, CI 33, RI 66)*. Contrary to taxa of CS-0 **[0]**, in *Loris*, *Nycticebus, Pithecia*, *Saimiri*, *Aotus*, *Macaca, Colobus, Papio*, *Cercopithecus*, and hominoids **[1]** the trapezius usually attaches onto the clavicle. *Propithecus* is coded as "?".
6. ***Trapezius*** *inserts onto ≥ lateral 1/3 of the clavicle (L 3, CI 33, RI 60)*. **[0]** Within those primates included in this study with an insertion of the trapezius onto the clavicle, an insertion onto less than the lateral 1/3 of the clavicle is usually found in *Macaca*, *Pithecia, Saimiri, Aotus*, *Colobus* and *Cercopithecus*. **[1]** In *Nycticebus*, *Loris*, *Papio* and hominoids the muscle goes to the lateral 1/3, or to more than the lateral 1/3, of the clavicle. *Propithecus* is coded as "?". **[-]** Inapplicable in taxa in which there is no insertion onto the clavicle (see character above).
7. ***Trapezius*** *does not insert onto the acromion (L 1, AUTAPOMORPHY)*. Contrary to taxa of CS-0 **[0]**, in *Tupaia* **[1]** the trapezius usually does not insert onto the acromion. *Tarsius* is coded as "?".
8. ***Trapezius*** *does not originate from the cranium (L 6, CI 16, RI 16)*. Contrary to taxa of CS-0 **[0]**, in *Rattus*, *Cynocephalus*, *Lemur*, *Propithecus*, *Tarsius*, *Callithrix*, and *Hylobates* **[1]** the trapezius is usually not directly originated from the cranium.
9. ***Sternocleidomastoideus*** *is hypertrophied (L 1, AUTAPOMORPHY)*. Contrary to taxa of CS-0 **[0],** in *Tarsius* **[1]** the sternocleidomastoideus is hypertrophied and has a peculiar, wide contact with its counterpart in the dorsal midline (of the nuchal/occipital region).
10. ***Constrictor pharyngis medius*** *has no pars ceratopharyngea (L 1, AUTAPOMORPHY)*. **[0]** In *Tupaia*, *Cynocephalus, Lemur*, *Propithecus*, *Tarsius*, *Aotus*, *Callithrix, Pithecia*, *Saimiri*, *Macaca*, *Papio*, *Cercopithecus*, *Colobus*, *Hylobates*, *Gorilla*, *Homo* and *Pan paniscus, Pan troglodytes*, the constrictor pharyngis medius is at least partially attached onto the greater horn of the hyoid bone, i.e. it has a pars ceratopharyngea. **[1]** In *Rattus* the constrictor pharyngis medius is not partially attached to the greater horn of the hyoid bone. *Pongo*, *Loris* and *Nycticebus* are coded as “?”.
11. ***Cricothyroideus*** *is differentiated into a pars recta and a pars obliqua (L 1, CI 100, RI 100)*. Contrary to taxa of CS-0 **[0]**, in the primate taxa included in this study (except *Nycticebus*, which is coded as "?") **[1]** the cricothyroideus usually has a distinct pars obliqua and a distinct pars recta.
12. ***Thyroideus transversus*** *is a distinct muscle (L 1, AUTAPOMORPHY)*. Contrary to taxa of CS-0 **[0]**, in *Hylobates* **[1]** there is often a distinct muscle thyroideus transversus (also designated in the literature as ‘thyroideus impar’), which lies on the ventral margin, and runs transversely to connect the posteroventromedial portion of the two sides, of the larynx.
13. ***Pterygopharyngeus*** *is not a distinct muscle* *(L 3, CI 33, RI 33).* Contrary to *Rattus*, *Cynocephalus* and *Hylobates* **[0]**, in *Tupaia*, *Lemur*, *Propithecus*, *Tarsius* and the non-hylobatid anthropoids included in this study **[1]** the pterygopharyngeus is either missing or fused with the constrictor pharyngis superior. *Loris* and *Nycticebus* are coded as “?”.
14. ***Thyroarytenoideus*** *is not differentiated into a pars superior and a pars inferior (L 1, CI 100, RI 100).* **[0]** In taxa of CS-0 the thyroarytenoideus is mainly divided into a more superior, and often lateral, pars superior - often also named ‘pars lateralis’ or ‘pars externa’ or ‘ventricularis’ -, and a more inferior, and often mesial, pars inferior – often also named ‘pars medialis’ or ‘pars interna’ or ‘vocalis’. **[1]** In *Callithrix*, *Aotus*, *Saimiri*, *Macaca*, *Papio*, *Cercopithecus*, *Colobus* and non-hylobatid hominoids the pars superior and pars inferior are not present as distinct structures. *Loris*, *Nycticebus*, *Pithecia* and *Hylobates* are coded as "?".
15. ***Arytenoideus*** ***obliquus*** *is a distinct muscle (L 1, CI 100, RI 100).* Contrary to taxa of CS-0 **[0]**, in *Pongo*, *Gorilla*, *Pan paniscus, Pan troglodytes* and *Homo* **[1]** the arytenoideus obliquus is often present as a distinct muscle.
16. ***Cricoarytenoideus* *posterior*** *does not meet its counterpart at the dorsal midline (L 4, CI 25, RI 66)*. Contrary to taxa of CS-0 **[0]**, in *Homo*, *Pan paniscus, Pan troglodytes, Gorilla, Papio, Colobus, Cercopithecus, Callithrix, Pithecia, Aotus* and *Saimiri* **[1]** the cricoarytenoideus posterior usually does not meet its counterpart at the dorsal midline. *Loris* and *Nycticebus* are coded as "?".

**Hypobranchial muscles**

1. ***Geniohyoideus*** *is fused to its counterpart in the midline (L 5, CI 20, RI 50)*. **[0]** In taxa of CS-0 the geniohyoideus usually lies very close to its counterpart at the ventral midline, but it is separated from it by fascia, a median raphe, and/or some other type of tissue, so that the muscles of two sides are not fused. **[1]** Such a fusion is usually found in *Tupaia*, *Lemur*, *Propithecus*, *Nycticebus*, *Papio*, *Colobus*, *Cercopithecus*, *Homo* and *Pan paniscus* and *Pan troglodytes*. *Loris* is coded as "?".
2. *Chondroglossus is present as a distinct bundle of the* ***hyoglossus*** *(L 3, CI 33, RI 60)*. Contrary to taxa of CS-0 **[0]**, in *Tupaia*, *Lemur*, *Propithecus*, *Pithecia*, *Macaca*, *Colobus*, *Cercopithecus*, and hominoids **[1]** the chondroglossus is usually present as a distinct bundle of the hyoglossus. *Papio*, *Nycticebus, Cynocephalus* and *Loris* are coded as "?".
3. ***Hyoglossus*** *is partially or completely fused with the* ***thyrohyoideus*** *(L 3, CI 33, RI 33)*. Contrary to taxa of CS-0 **[0]**, in *Cynocephalus*, *Aotus*, *Macaca* and *Papio* **[1]** the hyoglossus and thyrohyoideus are usually fused (partially or completely) to each other.
4. ***Styloglossus*** *originates from the stylomandibular ligament (ordered multistate character) (L 2, CI 100, RI 100).* In taxa of CS-0 **[0]** the styloglossus usually originates from the cranium and from ligamentous, cartilaginous or ossified structures of the hyoid apparatus such as the stylohyal ligament. In *Aotus*, *Callithrix*, *Pithecia* and *Saimiri* the styloglossus is at least partially originated from the stylomandibular ligament, the origin from this ligament being however less substantial in *Aotus* and *Pithecia* **[1]** than in *Callithrix* and *Saimiri* **[2]**. *Papio* and *Cercopithecus* are coded as "?".
5. ***Styloglossus*** *has a distinct oblique slip running anteroinferiorly to blend with the lateral portion of the* ***hyoglossus*** *(L 1, CI 100, RI 100)*. Contrary to taxa of CS-0 **[0]**, in *Pan paniscus, Pan troglodytes* and *Homo* **[1]** the styloglossus runs mainly longitudinally to insert onto the tongue but has a distinct oblique slip that runs anteroinferiorly at about 45º from the main body of the muscle to insert more inferiorly onto the lateral surface of the hyoglossus. *Pongo* is coded as "?".
6. ***Sternohyoideus*** *is divided into two bundles (L 1, AUTAPOMORPHY).* Contrary to taxa of CS-0 **[0]**, in *Cynocephalus* **[1]** the sternohyoideus has a configuration in which there is a belly that is mainly inserted onto the thyroid cartilage and that then gives rise to another belly that reaches the hyoid bone.
7. ***Sternohyoideus*** *does not contact nor lie against its counterpart for most of its length (L 2, CI 50, RI 50)*. Contrary to taxa of CS-0 **[0]**, in *Cynocephalus, Pan paniscus, Pan troglodytes* and *Homo* **[1]** the sternohyoideus usually does not contact, nor lies just next to, its counterpart for most of its length. *Hylobates*, *Nycticebus* and *Loris* are coded as "?".
8. *Anterior portion of* ***sternothyroideus*** *extends anteriorly to the posterior portion of the* ***thyrohyoideus*** *(L 4, CI 25, RI 25).* Contrary to taxa of CS-0 **[0]**, in *Rattus*, *Callithrix*, *Hylobates*, *Gorilla*, and *Pan paniscus* and *Pan troglodytes* **[1]** the main body of the sternothyroideus is usually extended anteriorly, so that its anterior portion is anterior to the posterior portion of the main body of the thyrohyoideus. *Pongo*, *Nycticebus* and *Loris* are coded as "?".
9. ***Omohyoideus*** *is not a distinct muscle (L 3, CI 33, RI 0).* Contrary to taxa of CS-0 **[0]**, in *Cynocephalus*, *Colobus* and *Cercopithecus* **[1]** the omohyoideus is usually not present as a distinct muscle. *Aotus* is coded as "?".
10. ***Omohyoideus*** *has an intermediate tendon (L 3, CI 33, RI 0).* Contrary to taxa of CS-0 **[0]**, in *Tupaia*, *Pan troglodytes* and *Homo* **[1]** the intermediate tendon of the omohyoideus is usually present. *Aotus* is coded as "?". **[-]** Inapplicable in *Cynocephalus, Colobus* and *Cercopithecus*, because the omohyoideus is usually not present as a distinct muscle.
11. ***Omohyoideus*** *occasionally has three bellies (L 1, CI 100, RI 100).* Contrary to taxa of CS-0 **[0]**, in at least some specimens of *Gorilla*, *Pan paniscus, Pan troglodytes* and *Homo* **[1]** the omohyoideus has three bellies (usually a superior belly, an inferomedial belly, and an inferolateral belly). *Aotus* is coded as "?". **[-]** Inapplicable in *Cynocephalus, Colobus* and *Cercopithecus*, because the omohyoideus is usually not present as a distinct muscle.

**Pectoral muscles**

1. ***Serratus anterior*** *and* ***levator scapulae*** *are separated (ordered multistate character) (L 2, CI 100, RI 100)*. **[0]** In *Rattus*, *Cynocephalus*, *Tupaia*, *Nycticebus, Propithecus*, *Lemur* and *Loris*, the serratus anterior and levator scapulae are deeply blended. In *Tarsius*, *Aotus, Callithrix, Pithecia*, *Saimiri*, *Macaca*, *Papio*, *Cercopithecus* and *Colobus* **[1]** the two muscles are less blended distally (at their insertion onto the scapula) than in taxa of CS-0, but more blended proximally than in hominoids **[2]**, in which the two muscles are well separated.
2. ***Rhomboideus major*** *and* ***rhomboideus minor*** *are not distinct muscles (L 4, CI 25, RI 57)*. Contrary to taxa of CS-0 **[0]**, in *Cynocephalus*, *Lemur*, *Propithecus*, *Loris*, *Nycticebus*, *Tarsius, Aotus, Pithecia*, *Saimiri* and non-human hominoids **[1]** the rhomboideus major and rhomboideus minor are not present as distinct muscles (i.e. these taxa have, instead, a single, undivided muscle rhomboideus). *Colobus* is coded as "?".
3. ***Rhomboideus occipitalis*** *is not a distinct muscle (L 3, CI 33, RI 50)*. Contrary to taxa of CS-0 **[0]**, in *Cynocephalus*, *Hylobates*, *Gorilla*, *Pan paniscus, Pan troglodytes* and *Homo* **[1]** the rhomboideus occipitalis is usually not present as a distinct muscle. *Propithecus* is coded as "?".
4. ***Levator scapulae*** *does not extend to C5 (L 1, CI 100, RI 100).* Contrary to taxa of CS-0 **[0]**, in hominoids **[1]** the origin of the levator scapulae usually does not extend posteriorly to C5. *Nycticebus* is coded as "?".
5. ***Levator claviculae*** *is not a distinct muscle (L 1, AUTAPOMORPHY)*. Contrary to taxa of CS-0 **[0]**, in modern humans **[1]** the levator claviculae is usually not present as distinct muscle.
6. ***Atlantoscapularis posticus*** *is a distinct muscle (L 1, AUTAPOMORPHY)*. Contrary to taxa of CS-0 **[0]**, *Tupaia* **[1]** has a muscle atlantoscapularis posticus. **[-]** This character is inapplicable in modern humans because the levator claviculae is usually not present as a distinct muscle.
7. ***Levator claviculae*** *inserts onto the clavicle (L 2, CI 50, RI 75)*. Contrary to taxa of CS-0 **[0]**, in *Colobus* and non-human hominoids **[1]** the levator claviculae is at least partially attached onto the clavicle. **[-]** This character is inapplicable in modern humans, because the levator claviculae is usually not present as a distinct muscle.
8. ***Levator claviculae*** *inserts deep to the insertion of the* ***trapezius*** *(L 3, CI 33, RI 77)*. Contrary to taxa of CS-0 **[0]**, in *Saimiri*, *Pithecia*, and the non-human catarrhines included in this study **[1]** the levator claviculae is usually deep to (covered either laterally or dorsally by) the trapezius. [-] This character is inapplicable in modern humans, because the levator claviculae is usually not present as a distinct muscle.
9. ***Levator claviculae*** *inserts onto a more medial portion of the clavicle (L 1, uninformative)*. In *Hylobates* **[0]** the insertion of the levator claviculae on the clavicle is considerably more lateral than in other non-human hominoids and in *Colobus* **[1]** (e.g., previous studies have reported a position index, from acromial end of clavicle, of 18.3 in *Hylobates*, contrary to, 38.2 and 38.4 in *Gorilla* and *Pan paniscus* and *Pan troglodytes*, respectively; also corroborated by our dissections of specimens of these three taxa and of the other terminal taxa included in this study). **[-]** This character is inapplicable in taxa where the levator claviculae is not present as a distinct muscle or where this muscle is present but does not insert onto the clavicle (see characters above).
10. ***Subclavius*** *originates from the third rib (L 1, AUTAPOMORPHY)*. Contrary to taxa of CS-0 **[0]**, in *Hylobates* **[1]** the origin of the subclavius often extends to rib 3 and/or its costal cartilage.
11. ***Pectoralis major*** *has no clavicular origin (L 4, CI 25, RI 57)*. Contrary to taxa of CS-0 **[0]**, in *Loris, Nycticebus*, *Aotus*, *Callithrix*, *Saimiri*, *Macaca*, *Cercopithecus* and *Pongo* **[1]** there is usually no clavicular origin of the pectoralis major. *Papio* and *Pithecia* are coded as "?".
12. *'****Pectoralis tertius****' a distinct muscle* *(L 1, AUTAPOMORPHY).* Contrary to taxa of CS-0 **[0]**, in *Rattus* **[1]** there is a distinct 'pectoralis tertius', which usually runs from the xiphoid process to the coracoid process of the scapula.
13. ***Pectoralis major*** *inserts onto the coracoid process (L 1, AUTAPOMORPHY).* Contrary to taxa of CS-0 **[0]**, in *Gorilla* **[1]** the abdominal head of the pectoralis major is usually at least partially inserted onto the coracoid process.
14. ***Pectoralis major*** *is blended with the* ***biceps brachii*** *(L 2, CI 50, RI 0).* Contrary to taxa of CS-0 **[0]**, in *Gorilla* and *Hylobates* **[1]** the abdominal head of the pectoralis major is usually blended with the biceps brachii.
15. ***Pectoralis major*** *has a pars capsularis in at least some specimens (L 1, CI 100, RI 100).* Contrary to taxa of CS-0 **[0]**, in *Aotus*, *Saimiri* and *Pithecia* **[1]** there is a distinct pars capsularis of the pectoralis major, which lies laterally to the main body of the muscle and is often separated from this main body by the cephalic vein. *Callithrix* is coded as “?”.
16. ***Pectoralis minor*** *inserts onto the coracoid process (L 6, CI 16, RI 37).* Contrary to taxa of CS-0 **[0]**, in *Rattus*, *Cynocephalus*, *Aotus*, *Saimiri*, *Hylobates*, *Pongo*, *Gorilla* and modern humans and *Pan paniscus* **[1]** the pectoralis minor is at least partially inserted onto the coracoid process. *Papio* and *Macaca* are coded as "?".
17. ***Pectoralis minor*** *inserts onto the clavicle (L 1, AUTAPOMORPHY).* Contrary to taxa of CS-0 **[0]**, in *Hylobates* **[1]** the pectoralis minor is often at least partially inserted onto the clavicle.
18. ***Panniculus carnosus*** *is not a distinct muscle (L 2, CI 50, RI 80).* Contrary to taxa of CS-0 **[0]**, *Pithecia* and hominoids **[1]** normally do not have a panniculus carnosus.
19. ***Deltoideus*** *is a single, continuous muscle (L 3, CI 33, RI 60).* **[0]** In taxa of CS-0 the deltoideus complex is usually divided into a deltoideus scapularis and a deltoideus acromialis et clavicularis, which can be further differentiated into two distinct muscles, the deltoideus acromialis and the deltoideus acromialis. **[1]** In the anthropoid primates included in this study, as well as in *Propithecus*, *Loris* and *Nycticebus* there is a single, continuous deltoideus muscle.
20. ***Teres minor*** *is not a distinct muscle (L 1,AUTAPOMORPHY).* Contrary to taxa of CS-0 **[0]**, in *Tupaia* **[1]** the teres minor is almost always absent as a distinct structure (being probably fused with the infraspinatus and/or the deltoideus scapularis).
21. ***Subscapularis*** *has a distinct pars posterioris (L 1, AUTAPOMORPHY).* Contrary to taxa of CS-O **[0]**, *Hylobates* **[1]** has a distinct, peculiar pars inferioris of the subscapularis, which is partially separated, medially, from the main, anterior portion of the muscle by a ridge of the scapula.
22. ***Latissimus dorsi*** *and* ***teres major*** *are fused (L 3, CI 33, RI 66).* **[0]** In taxa of CS-0 the distal tendon of the latissimus dorsi passes mainly dorsal to (and is not fused with) the distal tendon of the latissimus dorsi. **[1]** In *Tupaia*, *Pongo*, *Hylobates* and the Old World monkey genera included in this study **[1]** the distal tendons of the latissimus dorsi and of the teres major are usually partially or completely fused to each other, at their insertions onto the humerus.

**Arm muscles**

1. ***Dorsoepitrochlearis*** *has two distinct proximal heads* *originating from the* ***latissimus dorsi*** *and the* ***teres major*** *(L 1, AUTAPOMORPHY).*Contrary to taxa of CS-O **[0]**, *Tupaia* **[1]** has a peculiar configuration in which the dorsoepitrochlearis has two distinct proximal heads, one originating from the teres major and the other from the latissimus dorsi. **[-]** This character is inapplicable in modern humans because the dorsoepitrochlearis is usually not present as a distinct muscle (see character below).
2. ***Dorsoepitrochlearis*** *is not a distinct muscle (L 1, AUTAPOMORPHY).* Contrary to taxa of CS-O **[0]**, in modern humans **[1]** the dorsoepitrochlearis is usually not present as a distinct muscle.
3. ***Dorsoepitrochlearis*** *does not insert onto the olecranon process of the ulna (L 1, CI 100, RI 100).* Contrary to taxa of CS-0 **[0]**, in non-human hominoids **[1]** the dorsoepitrochlearis is usually mainly attached onto the medial epicondyle, the intermuscular septum and/or other surrounding structures, but not onto the olecranon process or the olecranon fascia. **[-]** This character is inapplicable in modern humans because the dorsoepitrochlearis is usually not present as a distinct muscle.
4. ***Dorsoepitrochlearis*** *is blended with the* ***biceps brachii*** *(L 1, AUTAPOMORPHY).* Contrary to taxa of CS-O **[0]**, in *Hylobates* **[1]** the dorsoepitrochlearis is usually deeply blended with the short head of the biceps brachii.
5. *Strong fascial connection between the* ***dorsoepitrochlearis*** *and the subscapular fascia and/or the scapula is present (L 1, CI 100, RI 100).* Contrary to taxa of CS-0 **[0]**, in *Aotus*, *Callithrix*, *Pithecia* and *Saimiri* **[1]** there is usually a strong fascial connection between the dorsoepitrochlearis and the subscapular fascia and/or scapula.
6. *Long head of* ***triceps brachii*** *is divided into a thinner, deep bundle and a broader, superficial bundle (L 1, AUTAPOMORPHY).* Contrary to taxa of CS-0 **[0**], in *Papio* **[1]** the long head of the triceps brachii is often peculiarly differentiated into a thinner, deep (ventral) bundle and a broader, superficial (dorsal) bundle.
7. ***Triceps brachii*** *has a posterior head (L 3, CI 33, RI 60).* **[0]** In taxa of CS-0 the triceps brachii is usually only differentiated into three main divisions (i.e. a long head, a lateral head, and a medial head, although some of this divisions may sometimes be partially differentiated into subdivisions). **[1]** In *Tupaia*, *Lemur*, *Propithecus*, *Loris, Nycticebus* and *Tarsius* the triceps brachii has a lateral head, a medial head, a long head, and also a distinct, peculiar posterior head.
8. *Long head of* ***triceps brachii*** *originates from half or more than half of the lateral border of the scapula (L 3, CI 33, RI 60).* Contrary to taxa of CS-0 **[0]**, in *Macaca*, *Colobus*, *Cercopithecus*, *Papio*, *Pongo*, and *Pan paniscus* and *Pan troglodytes* **[1]** the long head of the triceps brachii usually originates from half or more than half of the lateral border of the scapula. *Gorilla* is coded as "?".
9. *Strong fascial connection between the* ***triceps brachii*** *and the scapular spine and/or the axillary region is present (L 3, CI 33, RI 66).* Contrary to taxa of CS-0 **[0]**, in *Lemur*, *Loris*, *Nycticebus*, *Aotus*, *Callithrix*, *Saimiri* and *Pithecia* **[1]** there is usually a peculiar, strong fascial connection between the triceps brachii and the scapular spine and/or axillary region.
10. ***Brachialis*** *does not originate from the surgical neck of the humerus (L 3, CI 33, RI 60).* Contrary to taxa of CS-0 **[0]**, in *Cynocephalus*, *Loris*, *Nycticebus*, *Callithrix*, *Pithecia*, *Saimiri*, *Aotus*, *Papio*, *Colobus*, *Cercopithecus* and hominoids **[1]** the origin of the brachialis usually does not extend (i.e., it is distal) to the surgical neck of the humerus. *Macaca* is coded as “?”.
11. ***Biceps brachii*** *has no short head (L 1, CI 100, RI 100).* Contrary to taxa of CS-0 (in which both short and long heads of biceps brachii are present) **[0]**, in *Loris* and *Nycticebus* **[1]** the short head is usually missing.
12. *Short and long heads of* ***biceps brachii*** *are completely separated (L 1, AUTAPOMORPHY).* **[0]** Short and long heads of biceps brachii at least partially blended distally. **[1]** In *Tarsius* these two heads are often entirely separated. **[-]** Inapplicable in *Loris* and *Nycticebus*, because the short head of the biceps is usually missing.
13. *Short head of* ***biceps brachii*** *originates from the humerus (L 1, AUTAPOMORPHY).* **[0]** The short head of biceps brachii usually originates from the scapula. **[1]** In *Hylobates* the short head of the biceps brachii is usually at least partially originated from the humerus. **[-]** Inapplicable in *Loris* and *Nycticebus*, because the short head of the biceps is usually missing.
14. ***Biceps brachii*** *is blended with the* ***flexor digitorum superficialis*** *(L 1, AUTAPOMORPHY).* Contrary to taxa of CS-0 **[0]**, in *Hylobates* **[1]** the distal portion of the biceps brachii is deeply blended with the proximal portion of the flexor digitorum superficialis.
15. ***Biceps brachii*** *does not insert onto the ulna (L 2, CI 50, RI 50)*. **[0]** In *Cynocephalus* and *Tupaia* the biceps brachii usually inserts directly onto both the ulna and the radius. **[1]** *Rattus* and the primate taxa included in this study the biceps brachii usually inserts directly onto the radius, but not onto the ulna.
16. ***Biceps brachii*** *has no bicipital aponeurosis (L 4, CI 25, RI 57)*. **[0]** In taxa of CS-0 the biceps brachii is usually prolonged distally by a bicipital aponeurosis ('lacertus fibrosus' or 'lacertus carnosus'), which is commonly associated with the fascia covering forearm muscles such as the pronator teres. **[1]** In *Cynocephalus*, *Tupaia*, *Loris, Nycticebus*, *Tarsius*, *Callithrix*, *Saimiri*, *Pithecia*, *Papio, Colobus, Cercopithecus* and *Pongo* the bicipital aponeurosis is usually not present as a distinct structure. *Macaca* and *Aotus* are coded as "?".
17. *Bicipital aponeurosis (of* ***biceps brachii****) forms a 'lacertus carnosus' (L 1, AUTAPOMORPHY).* **[0]** Within those taxa with a bicipital aponeurosis, this structure usually forms a 'lacertus fibrosus' (i.e., it does not include fleshy muscular fibers). **[1]** In *Hylobates* the bicipital aponeurosis is usually at least partially fleshy, thus forming a 'lacertus carnosus' between the main body of the biceps and the flexor muscles of the forearm. *Macaca* and *Aotus* are coded as “?”. **[-]** This character is inapplicable in taxa that do not have a distinct bicipital aponeurosis (see character above).
18. *Coracobrachialis profundus is not present as a distinct head of the* ***coracobrachialis*** *(L 3, CI 33, RI 66).* **[0]** In taxa of CS-0 the coracobrachialis proprius (or 'medius') and coracobrachialis profundus (or 'brevis) are present as distinct structures, the coracobrachialis profundus being usually a short bundle running from the coracoid process to the proximal region of the humerus and often lying deep (dorsal) to the coracobrachialis proprius. **[1]** In hominoids and in *Rattus* and *Pithecia* the coracobrachialis profundus is usually absent as a distinct structure.

**Ventral (volar) forearm muscles**

1. ***Pronator quadratus*** *is not a distinct muscle (L 1, AUTAPOMORPHY).* Contrary to taxa of CS-0 **[0]**, in *Cynocephalus* **[1]** there is no distinct pronator quadratus.
2. ***Flexor pollicis longus*** *is a distinct muscle (L 2, CI 50, RI 0).* Contrary to taxa of CS-0 **[0]**, in *Hylobates* and modern humans **[1]** the flexor pollicis longus is usually present as a distinct muscle (i.e., with an independent fleshy muscle that sends a tendon to digit 1 only.
3. ***Flexor digitorum profundus*** *is not originated from the medial epicondyle of the humerus or from the common flexor tendon (L 2, CI 50, RI 75).* **[0]** In taxa of CS-0 the flexor digitorum profundus (and/or the flexor pollicis longus in *Hylobates*) usually originates from the medial epicondyle of the humerus and/or from the common flexor tendon associated with this epicondyle, as well as from the radius, ulna and/or interosseous membrane. **[1]** In *Macaca*, *Pongo*, *Gorilla*, *Pan paniscus, Pan troglodytes* and modern humans the origin of the flexor digitorum profundus (and of the flexor pollicis longus, in modern humans) is usually exclusively from the radius and/or ulna and, often, from the interosseous membrane. *Papio* and *Colobus* are coded as "?".
4. ***Flexor digitorum profundus*** *is not innervated by the ulnar nerve (L 1, CI 100, RI 100).* **[0]** In taxa of Cs-0 the flexor digitorum profundus is usually at least partially innervated by the ulnar nerve. **[1]** In *Macaca*, *Papio* and *Colobus* the muscle is usually not innervated by the ulnar nerve. *Aotus, Callithrix, Saimiri* and *Cercopithecus* are coded as “?”.
5. *Tendon of* ***flexor digitorum profundus*** *to digit 1 is vestigial or absent (L 4, CI 25, RI 0).* **[0]** In taxa of CS-0 the tendon of the flexor digitorum profundus to digit 1 (or the tendon of the flexor pollicis longus in *Hylobates* and *Homo*), is basically similar to the tendons of the flexor digitorum profundus to the other digits. **[1]** In *Colobus*, *Pongo*, *Gorilla* and *Pan troglodytes* the tendon to digit 1 is vestigial (i.e. it is markedly shorter and/or thinner than that of taxa of CS-0) or absent.
6. ***Flexor digitorum superficialis*** *originates from the radius (L 1, CI 100, RI 100).* Contrary to taxa of CS-0 **[0]**, in hominoids **[1]** the flexor digitorum superficialis usually partially originates from the radius.
7. ***Flexor digitorum superficialis*** *originates from the ulna (L 1, CI 100, RI 100).* Contrary to taxa of CS-0 **[0]**, in hominoids **[1]** the flexor digitorum superficialis usually partially originates from the ulna.
8. ***Flexor digitorum superficialis*** *inserts onto digit 5 (L 1, CI 100, RI 100).* **[0]** In *Rattus* and *Tupaia* the flexor digitorum superficialis usually inserts onto digits 2-4. **[1]**. In *Cynocephalus* and the primates included in this study this muscle is usually partially inserted onto digit 5.
9. ***Flexor digitorum superficialis*** *does not insert onto digit 2 (L 1, AUTAPOMORPHY).* Contrary to taxa of CS-0 **[0]**, in *Loris* **[1]** the flexor digitorum superficialis usually does not insert onto digit 2.
10. ***Palmaris longus*** *is hypertrophied (L 1, AUTAPOMORPHY)*. Contrary to taxa of CS-0 **[0]**, in *Cynocephalus* **[1]** the palmaris longus is hypertrophied.
11. ***Palmaris longus*** *is absent in > 5% of the cases (L 1, CI 100, RI 100)*. The palmaris longus is always, or almost always, present in the specimens of taxa of CS-0 **[0]**, but it is absent in more than 5% of the cases in specimens of *Gorilla*, *Pan paniscus, Pan troglodytes* and *Homo* **[1]***.*
12. ***Flexor carpi ulnaris*** *does not originate from the humerus (L 1, CI 100, RI 100).* **[0]** In taxa of CS-0 the flexor carpi ulnaris usually originates from the humerus (often from the medial epicondyle) and ulna (often from the coronoid process). **[1]** In *Cynocephalus* the muscle usually does not originate from the humerus. *Hylobates* is coded as “?".
13. ***Epitrochleoanconeus*** *is not a distinct muscle (L 3, CI 33, RI 60).* Contrary to taxa of CS-0 **[0]**, in *Loris, Nycticebus, Hylobates*, *Pongo*, *Gorilla* and modern humans **[1]** the epitrochleoanconeus is usually not present as a separate, well-defined muscle. *Rattus* is coded as "?".
14. ***Flexor carpi radialis*** *inserts onto the metacarpals II and III (L 2, CI 50, RI 83).* **[0]** In taxa of CS-0 the flexor carpi radialis usually inserts onto metacarpal III (as is usually the case in, e.g., *Rattus*) or metacarpal II (as is usually the case in, e.g., *Lemur* and *Propithecus*) or, in a few cases, onto other structures (as is usually the case in, e.g., *Cynocephalus*), but usually does not attach onto both the metacarpal II and III. **[1]** In *Tupaia*, *Tarsius*, *Pithecia*, *Aotus*, *Callithrix*, *Saimiri*, *Macaca*, *Papio*, *Cercopithecus*, *Colobus*, *Pongo*, *Gorilla*, *Pan paniscus, Pan troglodytes* and modern humans the muscle often inserts onto both metacarpals II and III. *Hylobates* is coded as "?".
15. ***Flexor carpi radialis*** *does not insert onto the metacarpal II or the metacarpal III (L 1, AUTAPOMORPHY).* Contrary to taxa of CS-0 **[0]**, in *Cynocephalus* **[1]** the flexor carpi radialis does not attach to either metacarpal II or metacarpal III, being instead usually exclusively attached onto the trapezium and/or the trapezoid. *Loris* is coded as "?".
16. ***Flexor carpi radialis*** *originates from the radius (L 2, CI 50, RI 50).* **[0]** In taxa of CS-0 the flexor carpi radialis usually has a bony origin from the humerus, but not from the radius. **[1]** In *Pongo*, *Gorilla* and *Pan paniscus, Pan troglodytes* the muscle has bony origins from at least the humerus and the radius. *Hylobates* is coded as "?".
17. ***Pronator teres*** *originates from the ulna (ordered multistate character) (L 2, CI 100, RI 100).* **[0]** In taxa of CS-0 the pronator teres usually has a bony origin from the humerus, and not from the ulna. Within hominoids a bony origin from the ulna (in addition to an origin from the humerus) is “frequent” (i.e. often but not usually, that is, present in < 50% of the cases) in *Hylobates* **[1]** and the rule (i.e. usually, that is, present in ≥ 50% of the cases) in *Pan paniscus, Pan troglodytes*, *Gorilla, Pongo* and modern humans **[2]**.

**Hand muscles**

1. ***Palmaris brevis*** *is not a distinct muscle (L 3, CI 33, RI 60).* Contrary to taxa of CS-0 **[0]**, in *Hylobates* and *Pongo* **[1]** the palmaris brevis is usually not present as a distinct muscle.
2. ***Palmaris brevis*** *is hypertrophied (L 1, AUTAPOMORPHY)*. Contrary to taxa of CS-0 **[0]**, in *Colobus* **[1]** the palmaris brevis is hyperthophied, having two peculiar, distinct, well-developed heads, one on the ulnar side of the hand and the other on the radial side of the hand. **[-]** This character is innaplicable in *Hylobates* and *Pongo* because the palmaris brevis is usually absent (see character above).
3. ***Flexor digitorum brevis manus*** *is a distinct muscle (L 2, CI 50, RI 0).* Contrary to taxa of CS-0 **[0]**, in *Cynocephalus* and *Tupaia* **[1]** the flexor digitorum brevis manus is usually present as a distinct structure.
4. ***Lumbricales*** *originate from thin* ***flexor digitorum profundus*** *tendons (L 1, AUTAPOMORPHY).* **[0]** In taxa of CS-0 the lumbricales are mainly originated directly from the main body of the insertion tendons of the flexor digitorum profundus. **[1]** In *Tarsius* these hand muscles are instead originated from peculiar, thin tendons that are, in turn, derived from the main body of the insertion tendons of the flexor digitorum profundus.
5. *There are frequently three, or instead usually seven,* ***lumbricales*** *(unordered multistate character) (L 2, AUTAPOMORPHIES).* **[0]** Taxa of CS-0 usually have four lumbricales, to digits 2-5. **[1]** In *Hylobates* the fourth lumbrical, i.e. the lumbrical going to digit 5, is frequently missing (i.e. it was missing in about 35% of the specimens dissected by others and by us). **[2]** *Cynocephalus* usually has a very unusual number of lumbricales, 7, which go to the radial and ulnar sides of digits 2, 3 and 4 and to the radial side of digit 5.
6. ***Lumbricales*** *originate from the dorsal surfaces of the tendons of the* ***flexor digitorum profundus*** *(L 1, AUTAPOMORPHY).* **[0]** In taxa of CS-0 the lumbricales are usually mainly originated from the ventral (palmar) surfaces of the tendons of the flexor digitorum profundus. **[1]** In *Hylobates* the lumbricales are mainly originated from the dorsal surfaces of these tendons.
7. ***Contrahentes digitorum*** *are missing (L 2, CI 50, RI 50).* Contrary to taxa of CS-0 **[0]**, in *Pongo*, *Gorilla* and modern humans **[1]** there are usually no contrahentes digitorum other than the adductor pollicis.
8. *Two sets of* ***contrahentes digitorum*** *are present (L 1, AUTAPOMORPHY).* Contrary to taxa of CS-0 **[0]**, *Tarsius* **[1]** usually has two sets of contrahentes. **[-]** Inapplicable in *Pongo*, *Gorilla* and modern humans because these in these taxa there are usually no contrahentes digitorum other than the adductor pollicis.
9. *There are more than two* ***contrahentes digitorum*** *(L 2, CI 50, RI 87).* **[0]** In taxa of CS-0 there are only two fleshy contrahentes (one to digit 2 and one to digit 5 in all these taxa except *Pan paniscus* and *Pan troglodytes*, in which the contrahentes usually go to digits 4 and 5 instead). **[1]** In *Tarsius, Aotus, Callithrix, Pithecia, Saimiri, Macaca, Papio, Cercopithecus, Colobus* and *Hylobates,* there are three (to digits 2, 4 and 5 in all these taxa except *Tarsius*: see characters above) or eight (two sets, to digits 2, 3, 4 and 5, in *Tarsius*: see character and below). **[-]** Inapplicable in *Pongo*, *Gorilla* and modern humans because these in these taxa there are usually no contrahentes digitorum other than the adductor pollicis.
10. *There are* ***contrahentes digitorum*** *to digits 2, 3, 4 and 5 (L 1, AUTAPOMORPHY).* Contrary to taxa of CS-0 **[0]**, *Tarsius* **[1]** has an unique condition in which there are contrahentes to digits 2-5 (see character above). **[-]** Inapplicable in *Pongo*, *Gorilla* and modern humans because these in these taxa there are usually no contrahentes digitorum other than the adductor pollicis.
11. ***Contrahentes digitorum*** *have a peculiar configuration (L 1, AUTAPOMORPHY).* Contrary to taxa of CS-0 **[0]**, in *Tarsius* **[1]** the contrahentes have a peculiar configuration, arising from the palm and passing to the proximal and distal phalanges of all the five digits and, in addition, passing from some of the digits to the proximal phalanx of an adjacent digit. **[-]** Inapplicable in *Pongo*, *Gorilla* and modern humans because these in these taxa there are usually no contrahentes digitorum other than the adductor pollicis.
12. ***Thin, deep additional slip of*** ***adductor pollicis*** *(****TDAS-AD****, or ‘****interosseous volaris primus of Henle’*** *of modern human anatomy) is present (ordered multistate character) (L 2, CI 100, RI 100).* **[0]** TDAS-AD not described in the literature nor found in any dissections. **[1]** In *Gorilla* and *Pan paniscus* and *Pan troglodytes* the TDAS-AD is present in some, but not in most, cases (i.e., in < 50% of the cases). **[2]** In modern humans the TDAS-AD is usually present (i.e. in ≥ 50% of the cases).
13. *Main body of* ***adductor pollicis*** *inserts onto much of metacarpal I (L 1, AUTAPOMORPHY).* **[0]** In taxa of CS-0 the most proximal area of insertion of the main body of the adductor pollicis (i.e. excluding the TDAS-AD, when this structure is present) is usually onto the proximal phalanx of the thumb, the metacarpophalangeal joint and/or the sesamoid bones lying near to this joint and/or eventually onto a small portion of the distal margin of metacarpal I, as well as eventually onto the distal phalanx of the thumb. **[1]** In *Hylobates* the adductor pollicis is directly inserted onto much of metacarpal I (i.e. functionally the muscle becomes an 'adductor' but also an 'opponens' of the thumb).
14. ***Adductor pollicis*** *has transverse and oblique heads* *(ordered multistate character) (L 2, CI 100, RI 100).* **[0]** In taxa of CS-0 the adductor pollicis is usually not differenciated into distinct transverse and oblique heads. In hominoids and the Old World monkeys *Papio*, *Colobus, Cercopithecus* and *Macaca* **[2]** the adductor pollicis had distinct oblique and transverse heads; in *Tarsius*, *Aotus*, *Saimiri* and *Pithecia* **[1]** the adductor pollicis is partly differentiated into oblique and transverse heads, particularly at its origin from metacarpal III and the contrahens fascia, but distally the two heads are blended (i.e. the differentiation is not as marked as in taxa scored as CS-2). *Callithrix* is coded as “?”.
15. ***Flexor brevis profundus 2*** *is not a distinct muscle (L 3, CI 33, RI 60).* Contrary to taxa of CS-0 **[0]**, in *Tupaia*, *Cynocephalus*, *Aotus*, *Callithrix*, *Pithecia* and *Saimiri* **[1]** the flexor brevis profundus 2 is usually not present as a distinct structure (i.e., it is either missing or completely fused with the part of the flexor brevis profundus 1 that forms the main body of the undivided ‘flexor pollicis brevis’ of these taxa).
16. ***Flexores breves profundi*** *are fused with the* ***intermetacarpales****, forming the* ***dorsal interossei*** *(L 5, CI 20, RI 60)*. Contrary to taxa of CS-0 **[0]**, in *Tupaia*, *Cynocephalus, Aotus*, *Callithrix*, *Pithecia*, *Saimiri*, *Hylobates*, *Pongo*, *Gorilla*, *Pan paniscus* and modern humans **[1]** the flexores breves profundi 3, 5, 6 and 8 are usually fused with the intermetacarpales 1, 2, 3 and 4, forming the dorsal interossei 1, 2, 3 and 4, respectively.
17. *Digit 4 is functional axis of* ***intermetacarpales/dorsal interossei*** *(L 1, CI 100, RI 100).* **[0]** In taxa of CS-0 the intermetacarpales/dorsal interossei are inserted onto the radial sides of digits 2 and 3 and the ulnar sides of digits 3 and 4 (i.e. their functional axis is digit 3). **[1]** In *Lemur*, *Propithecus, Nycticebus* and *Loris* the insertion is usually onto the radial sides of digits 2, 3 and 4 and the ulnar side of digit 4 (i.e. the functional axis is digit 4).
18. ***Interossei accessorii*** *are present (L 2, CI 50, RI 75).* Contrary to taxa of CS-0 **[0]**, *Nycticebus*, *Loris*, *Lemur*, *Propithecus* and *Hylobates* **[1]** usually have interossei accessorii. *Tarsius* is coded as "?".
19. ***Opponens pollicis*** *is a distinct muscle (L 2, CI 50, RI 75).* Contrary to taxa of CS-0 **[0]**, in *Pithecia*, *Aotus*, *Saimiri*, *Tarsius* and the strepsirrhine and catarrhine primates included in this study **[1]** there is usually a distinct opponens pollicis.
20. ***Opponens pollicis*** *reaches the distal portion of metacarpal I (L 2, CI 50, RI 80).* **[0]** In *Lemur*, *Propithecus*, *Tarsius*, *Aotus*, *Pithecia* and *Saimiri* the opponens pollicis usually inserts onto metacarpal I but does not reach the distal portion of this bone. **[1]** In *Loris*, *Nycticebus*, *Macaca*, *Papio*, *Colobus, Cercopithecus* and hominoids the opponens pollicis usually extends to the distal portion of metacarpal I, inserting partially, or exclusively, onto this distal portion and/or onto the phalanges of the thumb. **[-]** Innaplicable in taxa in which the opponens pollicis is usually not present as a distinct muscle (see character above).
21. ***Opponens pollicis*** *inserts onto the proximal and/or the distal phalanges of the thumb (L 1, AUTAPOMORPHY).* Contrary to taxa of CS-0 **[0]**, in *Hylobates* **[1]** the opponens pollicis is often partially inserted onto the proximal and/or distal phalanges of the thumb. **[-]** Innaplicable in taxa in which the opponens pollicis is not present as a distinct muscle or in which this muscle does not extend distally to the proximal portion of metacarpal I (see characters above).
22. ***Flexor digiti minimi brevis*** *is partly originated from the pisiform (L 2, CI 50, RI 0).* **[0]** In taxa of CS-0 the flexor digiti minimi brevis usually originates from the hamate, flexor retinaculum and/or surrounding structures such as the metacarpal V, but not from the pisiform. **[1]** In *Cynocephalus* and *Hylobates* the muscle is often partially originated from the pisiform.
23. ***Flexor digiti minimi brevis*** *inserts onto the middle phalanx and/or the distal phalanx of digit 5 (L 2, CI 50, RI 0).* Contrary to taxa of CS-0, in which the flexor digiti minimi brevis inserts mainly onto the metacarpophalangeal joint, the base or middle of the proximal phalanx and/or the extensor expansion of digit 5 **[0]**, in *Hylobates* and *Nycticebus* this muscle is often also inserted onto the middle phalanx and/or the distal phalanx of digit 5 **[1]**. *Loris* is coded as "?".
24. ***Opponens digiti minimi*** *is a distinct muscle (L 2, CI 50, RI 50).* Contrary to taxa of CS-0 **[0]**, in *Rattus* and the primates included in this study **[1]** the opponens digiti minimi is present as a distinct muscle.
25. ***Opponens digiti minimi*** *is divided into superficial and deep bundles (ordered multistate character) (L 2, CI 100, RI 100).* **[0]** Within those taxa with an opponens digiti minimi, in *Rattus* and the non-Catarrhini primates included in this study, the muscle is usually undivided. In hominoids **[1]** the muscle is usually slightly differentiated into superficial and deep bundles, while in *Papio*, *Colobus*, *Cercopithecus* and *Macaca* **[2]** the muscle is remarkably divided into a more superficial head and a deeper and broader head that, due to their peculiar differentiation, are often considered to be different muscles. **[-]** Innaplicable in *Tupaia* and *Cynocephalus*, in which the opponens digiti minimi is not present as a distinct muscle (see character above).
26. *Insertion of* ***opponens digiti minimi*** *extends proximally to the distal part of metacarpal V (L 1, CI 100, RI 100).* Contrary to *Rattus* **[0]**, in the primates included in this study **[1]** the insertion of the opponens digiti minimi extends to the distal portion of metacarpal V, the muscle being thus inserted along most, or the whole, proximodistal length of this bone. **[-]** Innaplicable in *Cynocephalus* and *Tupaia*, in which the opponens digiti minimi is not present as a distinct muscle.
27. ***Abductor digiti minimi*** *is divided into two well differentiated heads (L 1, CI 100, RI 100).* Contrary to taxa of CS-0 **[0]**, in *Macaca* and *Papio* **[1]** there is often a differentiation of the abductor digiti minimi into two heads, which in some cases are so markedly separated from each other that they are only connected through their distal tendons. *Homo* is coded as "?".

**Dorsal forearm muscles**

1. ***Brachioradialis*** *often inserts onto the trapezium (L 1, AUTAPOMORPHY).* Contrary to taxa of CS-0 **[0]**, in *Tarsius* **[1]** the brachioradialis is often inserted onto the trapezium.
2. ***Extensor carpi radialis longus*** *does not insert onto the metacarpal II (L 1, AUTAPOMORPHY).* Contrary to taxa of CS-0 **[0]**, in *Cynocephalus* **[1]** the extensor carpi radialis longus is not inserted onto the metacarpal II, being usually exclusively inserted onto the trapezium.
3. ***Brachioradialis*** *is not a distinct muscle (L 1, AUTAPOMORPHY).* Contrary to taxa of CS-0 **[0]**, in *Rattus* **[1]** the brachioradialis is usually missing.
4. ***Supinator*** *has no ulnar head (L 2, CI 50, RI 83)*. **[0]** In taxa of CS-0 the supinator has a single, humeral head (i.e. it mainly originates from the humerus and/or the elbow joint capsule and/or elbow ligaments). **[1]** *Loris, Nycticebus*, *Pithecia*, *Callithrix*, *Aotus*, *Saimiri*, *Macaca*, *Cercopithecus*, *Papio* and non-hylobatid hominoids usually have a distinct ulnar head of the supinator originating from the posterior portion of the ulna. *Hylobates* is coded as "?".
5. ***Extensor carpi ulnaris*** *does not originate from the ulna (L 2, CI 50, RI 85).* **[0]** In taxa of CS-0 the extensor carpi ulnaris originates from both the ulna and lateral epicondyle of the humerus. **[1]** In *Pithecia*, *Saimiri*, *Callithrix*, *Aotus*, *Macaca*, *Cercopithecus, Colobus* and *Papio* the extensor carpi ulnaris originates mainly from the lateral epicondyle and does not have a direct bony origin from the ulna. *Rattus* is coded as “?”.
6. ***Anconeus*** *is not a distinct muscle (L 1, AUTAPOMORPHY).* Contrary to taxa of CS-0 **[0]**, in *Hylobates* **[1]** the anconeus is usually not present as a distinct muscle.
7. ***Extensor digiti quarti*** *is not a distinct muscle (L 1, CI 100, RI 100).* Contrary to *Rattus* **[0]**, in the other taxa included in this study **[1]** the extensor digiti quarti is completely fused with the so-called ‘extensor digiti quinti proprius’, the two fused muscles forming the extensor digiti minimi, which often inserts onto digits 4 and 5, but may insert instead onto digits 3-5, or to digit 5 only (see characters below).
8. ***Extensor digiti minimi*** *inserts onto digit 5 only (L 3, CI 33, RI 60).* **[0]** In taxa of CS-0 the extensor digiti minimi (or the extensor digiti quarti plus the ‘extensor digiti quinti proprius’, in *Rattus*) inserts onto two or more digits - usually onto digits 4 and 5, but sometimes also onto digit 3, see character below. **[1]** In *Loris*, *Nycticebus*, *Hylobates*, *Gorilla*, *Pan paniscus, Pan troglodytes* and modern humans the extensor digiti minimi usually inserts onto digit 5 only.
9. ***Extensor digiti minimi*** *is partially inserted onto digit 3 (L 1, AUTAPOMORPHY)*. Contrary to taxa of CS-0 **[0]**, *Cynocephalus* **[1]** exhibits a peculiar condition in which the extensor digiti minimi inserts onto digit 3 (as well as onto digits 4 and 5; see characters above).
10. ***Extensor digiti minimi*** *originates from the radius (L 1, AUTAPOMORPHY)*. **[0]** In taxa of CS-0 the extensor digiti minimi (or the extensor digiti quarti plus the ‘extensor digiti quinti proprius’, in *Rattus*) has a bony origin from the lateral epicondyle of the humerus and/or, less often, from the ulna, but usually not from the radius. **[1]** In *Cynocephalus* the extensor digiti minimi has a bony origin from the lateral epicondyle but also from the radius.
11. ***Extensor indicis*** *usually inserts onto digits 1-3, digits 2-4 or digit 2 only (unordered multistate character) (L 4, CI 75, RI 83).* **[0]** In taxa of CS-0 the extensor indicis usually inserts onto digits 2 and 3. **[1]** In *Cynocephalus* the muscle is commonly inserted onto digits 1, 2 and 3. **[2]** In *Aotus*, *Saimiri*, *Pithecia*, *Callithrix* and *Hylobates* the muscle is commonly inserted onto digits 2, 3 and 4. **[3]** In *Gorilla*, *Pan paniscus, Pan troglodytes* and modern humans the muscle is commonly inserted onto digit 2 only. *Lemur* and *Propithecus* are coded as "?".
12. ***Extensor pollicis longus*** *is deeply blended with the* ***extensor indicis*** *(L 3, CI 33, RI 60).* Contrary to taxa of CS-0 **[0]**, in *Tupaia*, *Aotus*, *Callithrix*, *Pithecia*, *Saimiri*, and *Colobus* **[1]** the extensor pollicis longus is usually deeply blended with the extensor indicis, forming a mainly undivided fleshy belly (which is often designated as ‘extensor digitorum profundus‘).
13. ***Extensor pollicis longus*** *plus* ***extensor indicis*** *send two tendons to digit 2* *(L 2, CI 50, RI 75).*Within the tendons of the extensor pollicis longus and of the extensor indicis only one usually goes, in taxa of CS-0 **[0]**, to digit 2 (usually, one of the tendons of the extensor indicis). In *Saimiri*, *Callithrix*, *Aotus*, *Pithecia*, and *Colobus* **[1]** there are often two tendons going to digit 2, i.e. contrary to taxa of CS-0, in these genera the extensor pollicis longus sends a tendon not only to digit 1, but also to digit 2 (the other tendon that goes to digit 2 being part of the extensor indicis)**.** *Tarsius* is coded as "?".
14. ***Abductor pollicis longus*** *extends to the proximal phalanx of the thumb* *(L 2, CI 50, RI 0).* Contrary to taxa of CS-0 **[0]**, in gorillas and modern humans **[1]** there is usually a distal extension of the abductor pollicis longus, or of a structure differentiated from it (the extensor pollicis brevis, in modern humans: see character below), to the proximal phalanx of the thumb. *Rattus* and *Cynocephalus* are coded as “?”.
15. ***Extensor pollicis brevis*** *is a distinct muscle* *(L 2, CI 50, RI 0).* Contrary to taxa of CS-0 **[0]**, in modern humans and *Hylobates* **[1]** the extensor pollicis brevis is usually present as a distinct muscle.

**Table S1** Matrix of characters states of each of the 166 muscular characters listed above and used in the phylogenetic analysis, including for the first time *Pan paniscus* as a terminal taxon (differences between this species and *Pan troglodytes* are shown in red); "-" and "?" mean "inapplicable" and "missing", respectively (for more details, see Diogo & Wood ^2-3^).

0 0 0 0 0 0 0 0 0 0 1 1 1 1 1 1 1 1

0 1 2 3 4 5 6 7 8 9 0 1 2 3 4 5 6 6

1 0 0 0 0 0 0 0 0 0 0 0 0 0 0 0 0 6

*Rattus*  000000000000001000000000000000000000000100000-01010000000000000100000000000-0010001000000000000000000001001000000000000?00000000000000000000000--0010000010?00000000?0

*Cynocephalus* 1011100001--000000010010110000011101000000110-01000000000?1001101--01100000-00000010000000000000001000001-0100000010101001000010200000000011000--100--00100001011100?0

*Tupaia* 000001-10010000000000000110011011101000000100-10000010001100000001000000100-00000000001011000001000000001-0000000000000010000010000000000011000--000--0000000100001000

*Lemur* 101000010001000000000010110001011011000000110-01001010001100000000001000000-0000000000000000000101000001000000000010000000000000000000000000111000010100000001000?0000

*Propithecus* 10100001000100000000001111000101101100000011??01001010001100000000001?00000-0000000001000000000100000001000000000010000000000000000000000000111000010100000001000?0000

*Loris* 10100001?00000?00000001011000101101100000?1111000?10??0???0000??00001000000-010000000100000000010111--011-000000001100010?000000000000000000111100?1010000100110000000

*Nycticebus* 101000?1?00000000000001011000101101100000?1111000??0??0?1?0000??000010?0000-010000000100000000010111--011-000000001000010000000000000000000011110011010000100110000000

*Tarsius* 1000000100000010000110101100?1011011000000010-?1101010000000000000011000000-00000000000000000001000010011-00000000100000100000010001111001000?100001010100000100000?00

*Aotus* 101000111000001000010010111001-110110010001110000010110100110000???11000000-0100011001000000010001100001??0000?0001000001000000000001000011100100001010000110100021100

*Callithrix* 1010001101--001000010010111001-11011000010110-01001011010002000100010000000-01000?00010000000100011000011-0000?00010000010000000000010000?11000--001010000110100021100

*Pithecia* 1010001110?00010000100111110001110110010?011100000101?010101000000011000001-0?000100110000000100011000011-100000001000001000000000001000011100100001010000110100021100

*Saimiri* 1000001111--00100001001011100010101?001010111000001011010002000000011000001-01000110010000000100011000011-0000?0001000001000000000001000011100100001010000110100021100

*Macaca* 1000001?10?0001010010011110100111011111000111000001010000110000000010000001-010000?001001000000010?00001??000110001000001000000000001000020000110001211000110100000000

*Papio* 10000011101000101001001111010011101111100?111100001010011?1?000000010000001-0?0000?0010010000010101000011-000?10001000001000000000001000020000110001211000110100000000

*Cercopithecus* 10100??1101000101001001011010011101111100011100000101001110?00001--10000001-01000000010010000000101000011-0000?0001000001000000000001000020000110001210000110100000000

*Colobus* 10100??1101000100001001111000011101111100111100000101001110000001--1?000011100000000010010000000101000011-000?11001000001000010000001000020000110001210000110100001100

*Hylobates* 10100010100000100001001111010011101101100111110100110?00010000?1000211100110100010111101100110000010011101101000111000?1?0?11-0011001000120101111111110000?01110020001

*Pongo* 11---??010001110011100111100001110111110011111000?1010100100?00?00021010011101000010110010010000101000011-1001011110000110121-00001----0020100110001110000100100000000

*Gorilla* 1000001?1010001101010111110000111011111001111100001010110100000100121110011100011010110000010000?0100001001001011110010110120000001----1020100110001110000100110030010

*P.troglodytes* 1000001010100011010101111100001110111110011111000010101111001011011211100111000000001100000100001010000100100101111001001012000000000001020000110001110000100110030000

*P.paniscus* 1000001010100011010101111100001110111110011111000010101111001011001211100111000000101100000100001010000100100100111001001012000000000001020100110001110000100110030000

*Homo* 101000101010001101010111110000111011111001111100001010111100101001120111----0000001011000-1--00000100001001011001110010110020000001----202010011000111?000100110030011

**Table S2** Table summarizing the total number of mandibular, hyoid (not including the small facial, extrinsic muscles of the ear), branchial, hypobranchial, pectoral, arm, forearm and hand muscles in adults of the primate genera included in our cladistic analyses with numbers for *Pan paniscus* being included for the first time. Difference between this species and *Pan troglodytes* are given in red: note that the only difference regarding presence/absence of muscles between these two species is that the intermetacarpales 1-4 are not present as distinct muscles in bonobos, so these apes have in total four muscles less than common chimpanzees, thus having only one more muscle than modern humans.

| Muscles | *Lemur* | *Propithecus* | *Loris* | *Nycticebus* | *Tarsius* | *Pithecia* | *Aotus* | *Saimiri* | *Callithrix* | *Colobus* | *Cercopithecus* | *Papio* | *Macaca* | *Hylobates* | *Pongo* | *Gorilla* | *P. troglodytes* | *P. paniscus* | *Homo* |
| --- | --- | --- | --- | --- | --- | --- | --- | --- | --- | --- | --- | --- | --- | --- | --- | --- | --- | --- | --- |
| Mandibular | 8 | 8 | 8 | 8 | 8 | 8 | 8 | 8 | 8 | 7-8 | 7-8 | 8 | 8 | 8 | 7 | 8 | 8 | 8 | 8 |
| Hyoid (not extrinsic ear) | 25 | 24 | 24-26 | 26 | 24 | 22 | 23 | 21 | 22 | 24-25 | 26-27 | 25-26 | 26 | 26 | 26 | 26 | 26 | 26 | 27 |
| Branchial | 14-16 | 14-16 | 15-17 | 14-17 | 16-17 | 14-16 | 14-16 | 15-16 | 14-16 | 13-14 | 16 | 14-15 | 16 | 17 | 14-15 | 15-16 | 15 | 15 | 16 |
| Hypobranchial | 12 | 12 | 12-15 | 12-15 | 12 | 12-13 | 11-12 | 12 | 13 | 12 | 12 | 13 | 13 | 13 | 12-13 | 13 | 13 | 13 | 13 |
| Pectoral muscles | 17 | 15-16 | 16 | 16 | 17 | 15 | 16 | 16 | 17 | 16 | 17 | 17 | 17 | 14 | 15 | 14 | 14 | 14 | 14 |
| Arm muscles | 5 | 5 | 5 | 5 | 5 | 5 | 5 | 5 | 5 | 5 | 5 | 5 | 5 | 5 | 5 | 5 | 5 | 5 | 4 |
| Forearm muscles | 19 | 19 | 18 | 18 | 19 | 19 | 19 | 19 | 19 | 19 | 19 | 19 | 19 | 19 | 18 | 18 | 19 | 19 | 20 |
| Hand muscles | 30 | 30 | 30 | 34 | 32-36 | 22 | 22 | 22 | 21^31^ | 27 | 27 | 27 | 27 | 27 | 20 | 20 | 26 | 22 | 21 |
| **Total number** | **130-132** | **127-130** | **128-135** | **133-139** | **133-138** | **117-120** | **118-121** | **118-119** | **119-121** | **123-126** | **129-131** | **128-130** | **131** | **129** | **117-119** | **119-120** | **126** | **124** | **123** |
